# DCK confers sensitivity of DCTD-positive cancer cells to oxidized methylcytidines

**DOI:** 10.1093/procel/pwac028

**Published:** 2022-07-15

**Authors:** Ya-Hui Zhao, Wei Jiang, Hai Gao, Guo-Zheng Pang, Yu-Shuang Wu, Yuan-Xian Wang, Meng-Yao Sheng, Jia-Ying Xie, Wan-Ling Wu, Zhi-Jian Ji, Ya-Rui Du, Lei Zhang, Xiao-Qin Wang, Colum P Walsh, Hai Jiang, Guo-Liang Xu, Dan Zhou

**Affiliations:** Shanghai Key Laboratory of Medical Epigenetics, Laboratory of Cancer Epigenetics, Institutes of Biomedical Sciences, Medical College of Fudan University, Chinese Academy of Medical Sciences (RU069), Shanghai 200032, China; State Key Laboratory of Molecular Biology, Institute of Biochemistry and Cell Biology, Center for Excellence in Molecular Cell Science, Chinese Academy of Sciences, Shanghai 200031, China; Shanghai Key Laboratory of Medical Epigenetics, Laboratory of Cancer Epigenetics, Institutes of Biomedical Sciences, Medical College of Fudan University, Chinese Academy of Medical Sciences (RU069), Shanghai 200032, China; Center for Medical Research and Innovation, Shanghai Pudong Hospital, Fudan University, Shanghai 201399, China; Shanghai Key Laboratory of Medical Epigenetics, Laboratory of Cancer Epigenetics, Institutes of Biomedical Sciences, Medical College of Fudan University, Chinese Academy of Medical Sciences (RU069), Shanghai 200032, China; Shanghai Key Laboratory of Medical Epigenetics, Laboratory of Cancer Epigenetics, Institutes of Biomedical Sciences, Medical College of Fudan University, Chinese Academy of Medical Sciences (RU069), Shanghai 200032, China; Shanghai Key Laboratory of Medical Epigenetics, Laboratory of Cancer Epigenetics, Institutes of Biomedical Sciences, Medical College of Fudan University, Chinese Academy of Medical Sciences (RU069), Shanghai 200032, China; Shanghai Key Laboratory of Medical Epigenetics, Laboratory of Cancer Epigenetics, Institutes of Biomedical Sciences, Medical College of Fudan University, Chinese Academy of Medical Sciences (RU069), Shanghai 200032, China; Shanghai Key Laboratory of Medical Epigenetics, Laboratory of Cancer Epigenetics, Institutes of Biomedical Sciences, Medical College of Fudan University, Chinese Academy of Medical Sciences (RU069), Shanghai 200032, China; Department of Hematology, Huashan Hospital of Fudan University, Shanghai 200040, China; State Key Laboratory of Molecular Biology, Institute of Biochemistry and Cell Biology, Center for Excellence in Molecular Cell Science, Chinese Academy of Sciences, Shanghai 200031, China; State Key Laboratory of Molecular Biology, Institute of Biochemistry and Cell Biology, Center for Excellence in Molecular Cell Science, Chinese Academy of Sciences, Shanghai 200031, China; Department of Chemistry and Institutes of Biomedical Science, Shanghai Medical College, Fudan University, Shanghai 200032, China; Department of Hematology, Huashan Hospital of Fudan University, Shanghai 200040, China; Genomic Medicine Research Group, Biomedical Sciences, Ulster University, Coleraine BT52 1SA, United Kingdom; Centre for Research and Development, Region Gävleborg/Uppsala University, Gävle SE-801 87, Sweden; State Key Laboratory of Molecular Biology, Institute of Biochemistry and Cell Biology, Center for Excellence in Molecular Cell Science, Chinese Academy of Sciences, Shanghai 200031, China; Shanghai Key Laboratory of Medical Epigenetics, Laboratory of Cancer Epigenetics, Institutes of Biomedical Sciences, Medical College of Fudan University, Chinese Academy of Medical Sciences (RU069), Shanghai 200032, China; State Key Laboratory of Molecular Biology, Institute of Biochemistry and Cell Biology, Center for Excellence in Molecular Cell Science, Chinese Academy of Sciences, Shanghai 200031, China; Shanghai Key Laboratory of Medical Epigenetics, Laboratory of Cancer Epigenetics, Institutes of Biomedical Sciences, Medical College of Fudan University, Chinese Academy of Medical Sciences (RU069), Shanghai 200032, China; Center for Medical Research and Innovation, Shanghai Pudong Hospital, Fudan University, Shanghai 201399, China

## Dear Editor,

Cytidine analogs, such as decitabine (DAC) and cytarabine (ara-C), have been widely used in the clinical treatment for several cancer types, including myelodysplastic syndrome and acute myeloid leukemia (AML; Appelbaum et al. 1999; Saba 2007). However, drug resistance causing treatment failure and disease relapse is an unresolved problem to date. Certain cancer cells rely on the salvage enzymes cytidine deaminase (CDA) and dCMP deaminase (DCTD) to inactivate these cytidine derivative drugs by deamination (Jamieson et al. 1987; Ebrahem et al. 2012). It is imperative to develop new categories of chemotherapeutic nucleosides to overcome the drug resistance caused by such increased cellular deamination activity. The oxidized methylcytidines ­5-hydroxymethyl-2ʹdeoxycytidine (5hmdC) and 5-formy-2ʹdeoxycytidine (5fdC) have emerged as a new class of promising anticancer chemotherapeutic agents, especially for the above settings (Zauri et al. 2015). They were found to exert tumor-killing effect through CDA-directed deamination, which produces uridine derivatives 5hmdU and 5fdU that can be incorporated into genomic DNA, resulting in extensive DNA damage and subsequent cell cycle arrest and cell death (Zauri et al. 2015). The detrimental effects of deaminated derivatives of 5hmdC and 5fdC present a novel vulnerability in cancer cells bearing CDA-driven chemoresistance against the commonly used cytidine analogs. Nevertheless, high CDA expression is only found in a few cancer types (Zauri et al. 2015), which could limit the application of 5hmdC and 5fdC in cancer treatment. We reasoned that profiling the killing effects of 5hmdC and 5fdC on different cancer cells with various genetic backgrounds could help us discover new metabolic pathways and expand the therapeutic potential of 5hmdC and 5fdC.

**Figure 1.**
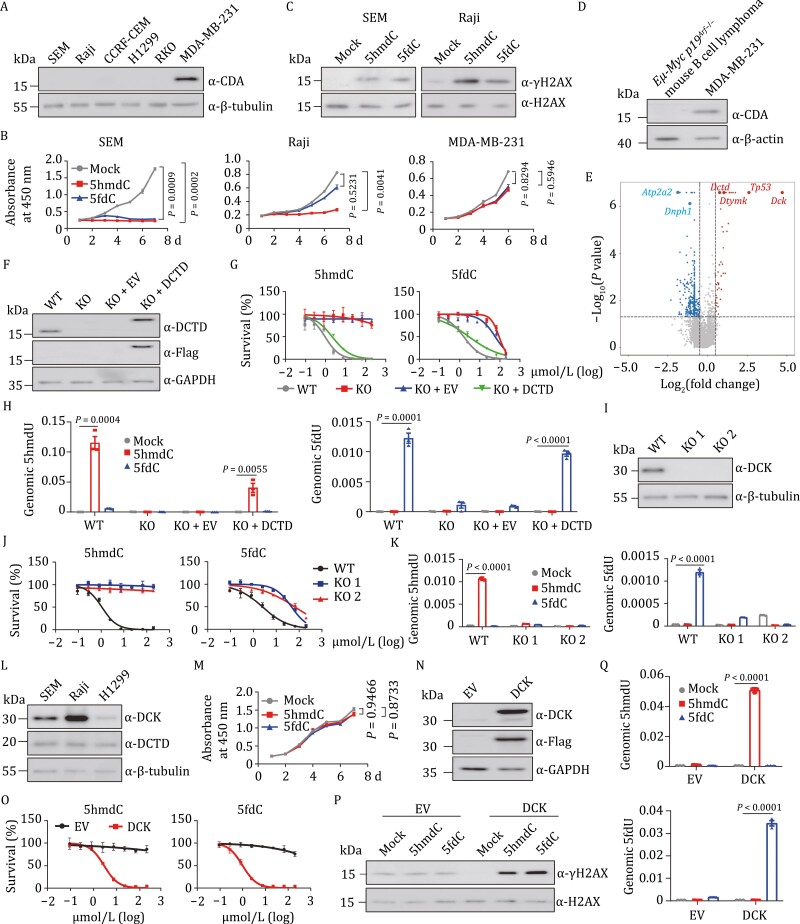
5hmdC and 5fdC inhibit proliferation of cancer cells expressing DCK and DCTD that promote the deamination of the two deoxynucleosides. (A) Western analysis of CDA in selected human cancer cell lines. (B) Growth curves of SEM (left), Raji (middle), and MDA-MB-231 cells (right) treated with PBS (mock, grey), 10 μmol/L 5hmdC (red), or 5fdC (blue) over a period of 7 days. Each dot represents the mean value of three replicates. Statistical differences were assessed between mock and 5hmdC- (or 5fdC-) treated groups by a two-way repeated ANOVA analysis. (C) Western blot analysis of γH2AX in SEM (left) and Raji (right) cells after a 2-day treatment with 10 μmol/L 5hmdC or 5fdC. (D) Western analysis of CDA in *Eµ-Myc p19*^*Arf*−/−^ and MDA-MB-231 cells. (E) Volcano plot displaying the results of MAGeCK analysis of the CRISPR screen. Red dots represent enriched genes and blue dots represent dropout genes in the surviving cells after two rounds of 5hmdC selection. 5hmdC was used at concentration of 200 µmol/L that killed 95% of cells. Significance is determined at *P*-value < 0.05, log_2_ (fold change) > 0.5. Sensitizing genes *Dck*, *Tp53*, *Dtymk,* and *Dtcd* and resistance genes *Atp2a2* and *Dnph1* are indicated. (F) Western blot showing the DCTD protein level in SEM cell line of wild type (WT), *DCTD* knockout (KO), KO infected with empty lentiviral vector (KO+EV) or with vector containing DCTD (KO+DCTD). (G) IC50 curves of 5hmdC (left) and 5fdC (right) in wild type (grey), *DCTD* KO (red), KO+EV (blue), and KO+DCTD (green) SEM cells. *X*-axis is the logarithmic transformed concentration of nucleosides. *Y*-axis denotes the proportion of live cells on day 4 at each nucleoside concentration. Each dot represents the average value of three technical replicates. (H) LC-MS/MS analysis of 5hmdU (left) and 5fdU (right) in the nucleoside pool hydrolyzed from genomic DNA of WT, KO, KO+EV, and KO+DCTD cell lines treated with 10 μmol/L 5hmdC (red) or 5fdC (blue). Levels of 5hmdU and 5fdU were normalized to the total amount of genomic dC in the indicated group (*n* = 3). (I) Western analysis of DCK in wild type (WT) and two *DCK* knockout (KO 1 and KO 2) cell lines of SEM. (J) IC50 curves of 5hmdC (left) and 5fdC (right) in WT and *DCK* KO SEM cell lines. (K) Abundance of 5hmdU (left) and 5fdU (right) measured by LC-MS/MS in genome of WT and two independent *DCK* KO SEM cell lines treated in triplicate with PBS (mock), 10 μmol/L 5hmdC or 5fdC. Total amount of genomic dC was used for normalization. (L) Western analysis of DCK and DCTD proteins in SEM, Raji, and H1299 cancer cells. (M) Growth curves of H1299 cells treated with PBS (mock, grey), 10 μmol/L 5hmdC (red) or 5fdC (blue) over a period of 7 days. (N) Western analysis confirming exogenous DCK expression in H1299 cells. EV, H1299 cells infected with empty lentiviral vector. DCK, H1299 infected with Flag-DCK lentiviruses. (O) IC50 curves of 5hmdC (left) and 5fdC (right) in DCK expressing cells. (P) Western analysis of γH2AX in DCK expressing cell lines after a 2-day treatment with PBS (mock), 10 μmol/L 5hmdC or 5fdC. (Q) LC-MS/MS analysis of the relative abundance of 5hmdU (top) and 5fdU (bottom) in the genome of DCK-expressing H1299 cells treated with 10 μmol/L 5hmdC (red) or 5fdC (blue). Cells transduced with empty lentiviral vector without DCK gene (EV) were used for comparison. The amount of genomic dC was used for normalization. Three replicates were used to calculate the mean value. For all panels: Significance is defined as *P* < 0.05. Error bar represents mean ± s.e.m. Mock, cell line treated with PBS.

We profiled half maximal inhibitory concentrations (IC50) of 5hmdC and 5fdC in 45 human cancer cell lines (Table S1). The survey showed that 10 cell lines were sensitive to 5hmdC, and 19 sensitive to 5fdC (IC50 < 50 μmol/L). By analyzing the mRNA level of *CDA* in these cell lines, we found that *CDA* is expressed in only two 5hmdC-sensitive and 14 5fdC-sensitive cell lines. Interestingly, a subset of cell lines lacking *CDA* expression, including SEM (a human acute lymphoblastic leukemia cell line) and Raji (a human B lymphoblastoid cell line), was strongly inhibited by either or both oxidized methylcytidines (Fig. 1A and Table S1), suggesting the presence of CDA-independent metabolic pathway(s) directing the cytotoxicity of 5hmdC and 5fdC. We analyzed the proliferation of SEM, Raji and MDA-MB-231 by supplementing 10 μmol/L 5hmdC or 5fdC in the culture media over a period of 7 days and found that the proliferation of SEM and Raji cell lines was significantly inhibited by 5hmdC or 5fdC (Fig. 1B), and that these two cell lines were much more sensitive to 5hmdC, 5fdC, or both, than MDA-MB-231, which expresses CDA (Fig. 1A). 5hmdC- and 5fdC-treated SEM and Raji cells showed an increased γH2AX signal (Fig. 1C), indicating that 5hmdC and 5fdC are able to induce DNA damage in the absence of CDA-directed deamination. Twenty-four hours of 5hmdC treatment resulted in S-phase arrest (Fig. S1A and S1B) and an increase in apoptosis (Fig. S1C and S1D) of SEM cells. Taken together, these data indicated that we have identified a group of cancer cells that are able to metabolize oxidized methylcytidines through CDA-independent pathway(s).

Due to the inability of cytidine monophosphate kinase 1 (CMPK1) to phosphorylate modified cytidine monophosphates, the direct incorporation of 5mdC, 5hmdC and 5fdC into the genome is prevented (Vilpo and Vilpo 1993; Zauri et al. 2015). A deamination step could thus be required for 5hmdC and 5fdC to kill CDA-null cancer cells. We performed a genome-wide CRISPR knockout screen in the *Eµ-Myc p19*^*Arf*−/−^ mouse B-cell lymphoma cell line, which was shown to be an ideal model system for analyzing gene–drug interactions (Jiang et al. 2011; Bruno et al. 2017) and does not express CDA (Fig. 1D). Cas9-expressing *Eµ-Myc p19*^*Arf*−/−^ cells were transduced with a genome-wide sgRNA library (Wang et al. 2017), followed by two rounds of 5hmdC treatment. To identify 5hmdC sensitizers, enriched sgRNAs in the surviving cells were ranked and compared with untreated control cells (Table S2). Bioinformatic analysis showed significant enrichment of sgRNAs for several genes, including *Dck*, *Tp53*, *Dtymk*, and *Dctd* (Fig. 1E), suggesting their critical roles in mediating the metabolic activation and cytotoxic functions of 5hmdC. Among these genes, *Dck* is the highest-ranking hit which converts 5hmdC to 5hmdCMP, while *Dctd* encodes a nucleotide deaminase which may carry out the deamination step to generate cytotoxic 5hmdUTP. Dropout analysis also revealed 5hmdC-resistance genes, such as ATPase sarcoplasmic/endoplasmic reticulum Ca^2+^ transporting 2 (*Atp2a2*) and 2ʹ-deoxynucleoside 5ʹ-phosphate N-hydrolase 1 (*Dnph1*), a nucleotide salvage factor recently found to be responsible for removing 5hmdUMP (Fugger et al. 2021). Thus, we show that the metabolism of 5hmdC is subjected to multiple levels of regulation in cancer cells.

Previous work showed that extracts from unfertilized eggs and embryos of sea urchins and chicks containing DCTD can catalyze the deamination of 5hmdCMP (Scarano 1960a, 1960b; Maley and Maley 1966). In human cancer cells, 5hmdCMP and 5fdCMP can be generated by DCK through the phosphorylation of 5hmdC and 5fdC (Zauri et al. 2015), thus providing potential substrates for DCTD. We therefore analyzed DCTD’s deaminase ability on 5hmdCMP and 5fdCMP using human DCTD and CDA recombinant proteins (Fig. S2A). The *in vitro* deamination assay indicated that dCMP could be converted to dUMP by DCTD and CDA (Fig. S2B, left). Both 5hmdCMP and 5fdCMP could serve as substrates for DCTD- and CDA-directed deamination, as indicated by the detection of 5hmdU and 5fdU nucleosides (Fig. S2B, middle and right). Notably, the deamination rate of 5hmdCMP by DCTD was markedly higher than that of 5fdCMP, indicating that 5hmdC is more efficiently converted into active form by DCTD than 5fdC is. This is in contrast to CDA-directed deamination activity, which converts 5fdC (Zauri et al. 2015) and 5fdCMP more efficiently than 5hmdC and 5hmdCMP. Our finding is consistent with the observation that 5hmdC exerts a stronger inhibitory effect than 5fdC does on SEM and Raji cells (Fig. 1B), and that a greater proportion of cell lines sensitive to 5fdC express *CDA* than not (14/19), while most 5hmdC-sensitive cells have no *CDA* expression (8/10) (Table S1).

To determine whether DCTD is the key factor mediating the sensitivity to 5hmdC and 5fdC, we profiled the IC50 of these two cytidine variants in *DCTD* KO SEM and Raji cells (Figs. 1F and S3A–C). Deficiency of *DCTD* completely abolished the inhibitory effect of 5hmdC and markedly reduced the susceptibility to 5fdC in SEM and Raji cells (Figs. 1G and S3D), suggesting the existence of other metabolic pathway(s) mediating the cytotoxicity of 5fdC. Re-expression of DCTD in *DCTD* KO SEM and Raji cells fully restored the inhibitory effects of oxidized methylcytidines in SEM and Raji cells (Figs. 1G and S3D). The differential killing effect among examined cell lines was not due to a difference in the uptake of the nucleosides as the free 5hmdC and 5fdC nucleosides were detected in the cellular metabolites of both wild-type and *DCTD*-deficient SEM cells (Fig. S4A and S4B). However, the deamination products of 5hmdCMP and 5fdCMP, respectively measured in the form of 5hmdU and 5fdU nucleosides in LC-MS/MS, were detected only in the cellular metabolites of KO cells rescued with Flag-DCTD and wild-type cells (Fig. S4C and S4D). Furthermore, incorporation of 5hmdU (or 5fdU) into the genome could be confirmed in DCTD-expressing cells upon treatment with 5hmdC (or 5fdC) (Fig. 1H). The results for Raji cells were similar to those for SEM cells (data not shown). These results suggest that the cytotoxicity and deamination of 5hmdC and 5fdC rely on the presence of DCTD in SEM and Raji cell lines.

The substrates of DCTD, specifically 5hmdCMP and 5fdCMP, are presumably provided by DCK. To confirm that DCK is required for the cytotoxicity of 5hmdC and 5fdC, we generated *DCK* KO SEM and Raji cell lines (Figs. 1I and S5A–C). Indeed, deficiency of *DCK* significantly compromised the inhibitory effects of 5hmdC and 5fdC in the two cell lines (Figs. 1J and S5D). Consistent with the proposed role of DCK, SEM cells lacking DCK had similar cellular uptake of 5hmdC and 5fdC (data not shown), but became devoid of 5hmdU and 5fdU to a lesser degree in the free cellular metabolite pools (Fig. S6), as well as in their incorporation into the genome (Fig. 1K). Interestingly, the proliferation of H1299 (a human non-small cell lung carcinoma cell line) and RKO (a human colon carcinoma cell line) cell lines was not affected by 10 μmol/L 5hmdC or 5fdC, even though both DCK (albeit at a lower level) and DCTD were expressed in these two cell lines (Figs. 1L, 1M, S7A and S7B). To test whether there was a dosage effect of DCK, we overexpressed it in H1299 and RKO cells (Figs. 1N and S7C). This led to a pronounced inhibition of cell growth by 5hmdC or 5fdC treatment (Figs. 1O and S7D). Similar to SEM and Raji cells, DCK-overexpressing H1299 cells exhibited an increased γH2AX signal after 5hmdC or 5fdC treatment (Fig. 1P). As expected, there was also a substantial increase of 5hmdU (or 5fdU) in the genome of DCK-overexpressing H1299 cells (Fig. 1Q). These data demonstrate that abundant expression of DCK is one of the key determinants of the cytotoxicity of these two oxidized methylcytidines, which is consistent with *Dck* being the highest-ranking hit in the CRISPR screening. Nevertheless, other potential factors, such as the cellular uptake capacity mediated by nucleoside transporters and expression of nucleoside hydrolases that prevent the genomic incorporation of modified nucleotides, should also be considered when assessing the cytotoxicity of these two compounds.

To evaluate the anti-tumor effect of 5hmdC, we established a subcutaneous xenograft model using wild type and *DCTD* knockout Raji lymphoma cells in BALB/c nude mice. We first assessed the *in vivo* metabolism of 5hmdC by tracking the peripheral blood levels of 5hmdC and 5hmdU upon intraperitoneal administration. 5hmdC rapidly increased upon intraperitoneal injection (50 mg/kg), reaching about 600 μmol/L in 10 min and being eliminated from peripheral blood within 4 h (Fig. S8A). 5hmdU, the deamination product of 5hmdC, was detected at a much lower level, manifesting a slightly slower accumulation and elimination as compared with 5hmdC (Fig. S8A).

**Figure 2.**
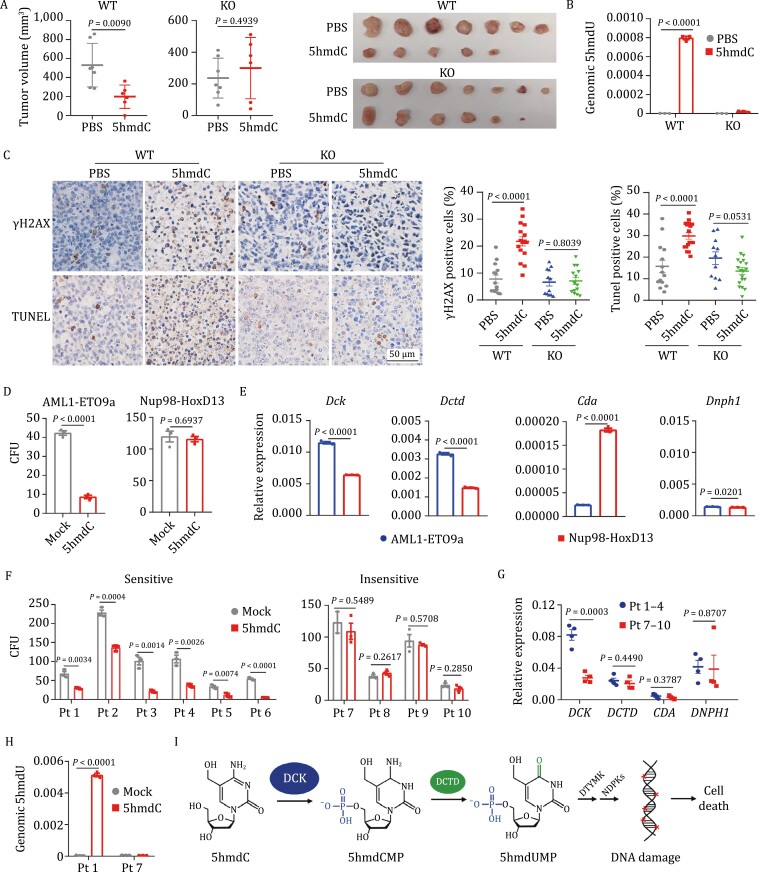
5hmdC inhibits the growth of subcutaneous tumors and colony formation of leukemia cells expressing DCK and DCTD. (A) Tumor volumes of wild-type (WT) and *DCTD* knockout (KO) Raji cells transplanted in BALB/c nude mice treated with PBS (*n* = 7) or 5hmdC (*n* = 6) at 50 mg/kg per day for 11 days. Dissected tumor specimens from each group are shown to the right. (B) Quantification of 5hmdU content by LC-MS/MS (normalized to genomic level of dC) in the genome of WT and *DCTD* KO xenograft tumors grown in mice treated with 5hmdC or PBS control. Three biological replicates were used to calculate the mean value. (C) Representative immunohistochemistry (IHC) images of γH2AX and TUNEL staining in WT and *DCTD* KO tumors from recipient mice treated with 5hmdC for 11 days. Quantification analysis of IHC staining is shown to the right. *Y*-axis denotes the percentage of cells stained positive for γH2AX or TUNEL. (D) Colony formation assay of bone marrow cells derived from mouse leukemia models harboring the fusion oncoproteins AML1-ETO9a or Nup98-HoxD13. Cells expressing the indicated fusion genes were cultured in the presence of 10 μmol/L 5hmdC in triplicate over a period of 1 week. *Y*-axis denotes the counts of colony in each treatment group. (E) mRNA levels of *Dck*, *Dctd*, *Cda*, and *Dnph1* measured by RT-qPCR in the bone marrow cells derived from mouse leukemia models harboring the indicated fusion oncoproteins. *Actb* was used as the internal control. (F) Colony formation assay of bone marrow cells derived from ten different leukemia patients. Bone marrow cells were cultured in triplicate in the presence of 20 μmol/L 5hmdC over a period of 2 weeks and the numbers of colonies were scored. *Y* axis denotes the colony counts in each treatment group. (G) mRNA levels of *DCK*, *DCTD*, *CDA*, and *DNPH1* measured by RT-qPCR in the untreated bone marrow cells derived from patient samples which were sensitive (Pt 1–4, blue) and insensitive (Pt 7–10, red) to 5hmdC. Each dot represents a patient sample. *GAPDH* was used as the internal control. (H) LC-MS/MS analysis of 5hmdU in the genome extracted from 5hmdC- treated bone marrow cells of patients 1 and 7. Levels of 5hmdU were normalized to the total amount of genomic dC in indicated groups. (I) A working model for 5hmdC metabolism. For all panels: Significance is defined as *P* < 0.05. Error bar represents mean ± s.e.m. Mock, cells treated with PBS. Pt, patient.

Wild-type and *DCTD* KO Raji cells were then subcutaneously inoculated into each flank of BALB/c nude mice. After a week, mice were randomized and received PBS or 5hmdC daily (50 mg/kg, intraperitoneal injection) for 11 days. Xenograft tumor growth of wild-type Raji cells was significantly restricted by 5hmdC treatment while that of *DCTD*-deficient cells was not affected (Fig. S8B). Consistently, treatment with 5hmdC significantly reduced the volumes and weight of wild-type Raji xenografts but had little effect on *DCTD* KO tumors (Figs. 2A and S8C). As expected, the genomic level of 5hmdU significantly increased in 5hmdC-treated wild type but not *DCTD* KO tumors (Fig. 2B). Significantly increased staining by γH2AX and TUNEL was observed in wild-type tumors treated with 5hmdC (Fig. 2C).

No evident alterations were noticed on behavior, overall body weight (data not shown) and spleen size in 5hmdC-treated animals (Fig. S8D). The percentages of Lin^−^Sca-1^+^c-Kit^+^ (LSK) hematopoietic stem/progenitor cells and Gr-1^+^Mac-1^+^ myeloid cells in the bone marrow of 5hmdC-treated mice were not changed either (Fig. S8E–H). The concentrations of white blood cells, red blood cells, platelets, and hemoglobin were not affected by 5hmdC (Fig. S8I–L). Taken together, these results demonstrate that 5hmdC can be safely applied *in vivo*, and that it can efficiently eliminate lymphoma cells in a DCTD-dependent manner.

To further evaluate the therapeutic potential of 5hmdC and 5fdC in hematopoietic malignancies, we examined their effects on the colony-forming ability of bone marrow cells derived from two different mouse leukemia models, harboring the fusion oncoproteins AML1-ETO9a (Wang et al. 2011) or Nup98-HoxD13 (NHD13) (Lin et al. 2005), respectively. We found that colony formation in AML1-ETO9a was markedly inhibited in the presence of 10 µmol/L 5hmdC, while no obvious effects were observed in NHD13 cells (Fig. 2D). Treatment with 10 µmol/L 5fdC did not exhibit an inhibitory effect on any of these cell lines (Fig. S9A). Since the conversion of 5hmdC to 5hmdU for genomic incorporation depends on the expression of *Dck, Dctd, Cda*, and *Dnph1* (Fugger et al. 2021), we examined their expression in these cells, and the results suggested that the high expression of *Dck* and *Dctd* in AML1-ETO9a cells correlated well with the inhibitory effect of 5hmdC on the colony formation of these leukemia cells (Fig. 2E).

We next sought to assess the anticancer efficacy of 5hmdC in human leukemia in relation to *DCTD* and *DCK* expression in various patient samples. We first examined the adverse effects of 5hmdC on human hematopoietic stem cells (CD34^+^) derived from normal cord blood using colony formation assay. Treating hematopoietic stem cells (CD34^+^) with up to 100 μmol/L 5hmdC exerted little inhibitory effect on their colony forming abilities (Fig. S9B). Then, we tested the bone marrow cells derived from 10 leukemia patients, harboring commonly found mutations, such as *DNMT3A*, *IDH2* and *TET2* (Table S3). Notably, treatment with 20 µmol/L 5hmdC resulted in a pronounced reduction of colonies in the samples of patients 1–6 but not in those of patients 7–10 (Fig. 2F). *DCK*, *DCTD*, *CDA*, and *DNPH1* expression levels were examined in the untreated cells (mock) derived from all patient samples except patients 5 and 6 due to insufficient cell numbers. Importantly, *DCK* was found to be highly expressed only in the bone marrow cells of patients 1–4 but not in those of patients 7–10, whereas no significant difference was observed in the expression of *DCTD, CDA* and *DNPH1* between patients 1–4 and patients 7–10 (Fig. 2G). Further analysis by LC-MS/MS demonstrated that 5hmdC treatment in patients 1 and 7 did not alter the genomic level of 5hmdC (data not shown), but rather resulted in a significant increase of genomic 5hmdU in patient 1 highly expressing *DCK* (Fig. 2H). Due to limited samples, the effect of treatment with 5fdC was tested only in the samples from patients 1, 2, and 7. Colony formation was not significantly affected by 20 µmol/L 5fdC (Fig. S9C). Taken together, these results suggest that DCTD plays a preeminent role in the metabolic activation of 5hmdC in human leukemia, and that high DCK expression can be utilized as a potential biomarker for therapeutic use of 5hmdC in human leukemia.

In summary, our study unveils a new intracellular metabolic pathway of 5hmdC and 5fdC reliant on DCK and DCTD, which mediates the anti-tumor effects and expands the therapeutic potential of these compounds. This pathway produces 5hmdCMP and 5fdCMP, which give rise to 5hmdUMP and 5fdUMP. Both 5hmdUMP and 5fdUMP undergo phosphorylation by deoxythymidylate kinase (DTYMK) and nucleoside diphosphate kinases (NDPKs), generating 5hmdUTP and 5fdUTP that can be incorporated into the genome by DNA polymerase and eventually result in DNA damage and cell death (Fig. 2I). According to the GEPIA database, DCTD is ubiquitously expressed across various tissues while DCK is upregulated in at least 11 tumor types (Fig. S10). In contrast, expression of CDA varies extensively and is subject to up- or downregulation in different tumor types (Fig. S10). This is in line with our observation that 5hmdC, the preferred substrate of DCTD, but not 5fdC, specifically inhibits colony formation in leukemia patient-derived bone marrow cells with high DCK expression. In addition, DCK upregulation in tumor cells may indicate a minimized 5hmdC toxicity for normal tissues in which DCK expression is relatively low, consistent with the lack of discernible adverse effects on cord blood-derived CD34^+^ cells and in mice administered with 5hmdC. As the abundance of DCK determines the tumor killing effect of oxidized methylcytidines in a DCTD-dependent manner, expression analysis of components of the DCK-DCTD axis in patients would provide a useful biomarker in cancer therapeutic applications of 5hmdC and 5fdC.

## Supplementary information

The online version contains supplementary material available at https://doi.org/10.1093/procel/pwac028.

pwac028_suppl_Supplementary_Material_S1

pwac028_suppl_Supplementary_Material_S2

## Footnotes

This work was supported by the National Natural Science Foundation of China (Nos. 32000894, 32000420), the Ministry of Science and Technology of China (No. 2018YFA0800302), the China postdoctoral Science Foundation (No. 2019M651357), the Shanghai Sailing Program (No. 18YF1412200), the Chinese Academy of Sciences Interdisciplinary Innovation Team (No. JCTD-2018-14), the Natural Science Foundation of Shanghai (NO. 20ZR1408000), Fudan University Start-up Research Grant (Nos. IDH1340038, IDH1340045, IDH1340046, IDH1340059) and the Chinese Academy of Medical Sciences (CAMS) Innovation Fund for Medical Sciences (CIFMS, No. 2019-I2M-5-077).

We acknowledge B.-B. Zhou and D.-P. Yang for the discussions and critical reading of the manuscript; L. Wang, X.-X. Huang for providing mouse leukemia cells and human cord blood cells; S.-Q. Rong for assistance with protein purification and deamination assay.

The authors declare no conflicts of interest and competing interests.

All experimental work was approved in advance by the Ethics Review Committee for Animal Experimentation of Fudan University.

All data and material are available upon request.

D. Zhou, G.-L. Xu and H. Jiang conceived and supervised the project. D. Zhou, Y.-H. Zhao and H. Gao designed the study, performed the most experiments and wrote the manuscript. W. Jiang performed CRISPR-Cas9 knockout screen. G.-Z. Pang carried out the quantitative real-time PCR and performed the biochemical assays. Y.-S. Wu performed the protein purification. Y.-X. Wang assisted with the tumor xenograft model and colony formation assay. M.-Y. Sheng contributed to patient sample collection. J.-Y. Xie conducted the flow cytometry analysis of cell cycle and apoptosis. X.-Q. Wang and W.-L. Wu provided human primary leukemia samples. L. Zhang performed the mass spectrometry analysis of nucleosides. Z.-J. Ji and Y.-R. Du provided critical preliminary results and biochemical data. C.P. Walsh assisted with experimental design and manuscript writing.
