# Supplementary material for: DCK confers sensitivity of DCTD-positive cancer cells to oxidized methylcytidines
Source: Protein Cell. 2022 Jul 15;14(7):534–9. doi: 10.1093/procel/pwac028 (PMC10305735; doi:10.1093/procel/pwac028)
Supplement: pwac028_suppl_Supplementary_Material_S1 [file pwac028_suppl_supplementary_material_s1.docx]

**Supplementary Figures and Figure Legends**

**
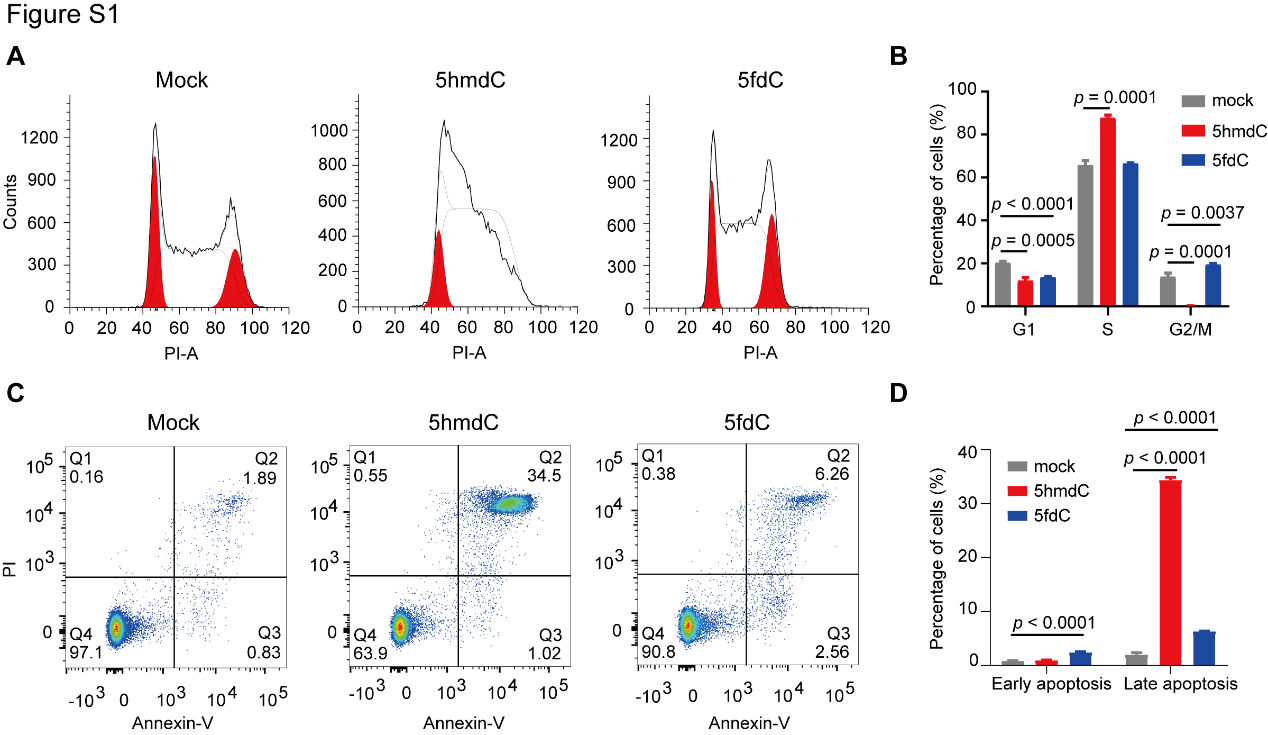
 Fig. S1. Administration of 5hmdC and 5fdC induces cell cycle changes and apoptosis.**

(A-B) Cell cycle analysis by propidium iodide assay in SEM cell lines treated with 10 μM 5hmdC or 5fdC for 24 hours. Shown are representative plots of SEM cells (*A*) and quantification of cells at each stage using three biological replicates (*B*). Mock, cell line treated with PBS. (C-D) Apoptosis analysis of SEM cells treated with 10 μM 5hmdC or 5fdC analyzed by Annexin V-PI FACS assay. Shown are representative plots (C) and quantification of early (Q3) and late (Q2) apoptotic cells (D) of indicated group after one day treatment. Three biological replicates were used to calculate the mean value. Quantifications are expressed as mean ± standard error (mean). Significance is defined as *p* < 0.05.


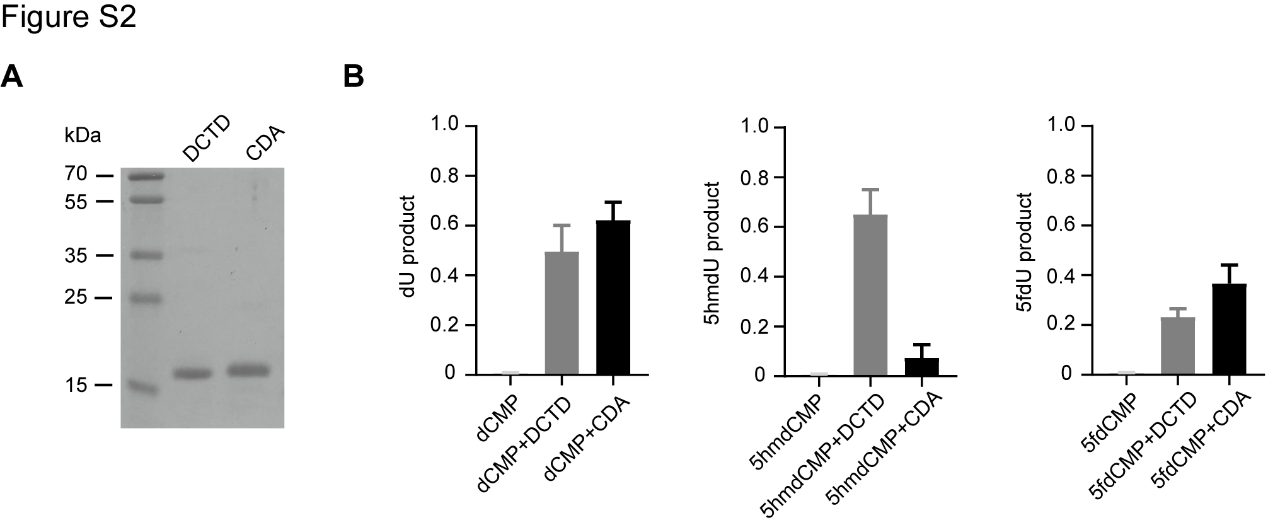


**Fig. S2. Characterization of the deamination activities of DCTD and CDA on 5hmdC and 5fdC monophosphates.**

(A) Coomassie-stained SDS-PAGE gel image of recombinant human DCTD and CDA proteins purified from *E. coli*. (B) *In vitro* deamination assay and LC-MS/MS analysis of DCTD and CDA-catalyzed deamination reactions. The C-, 5hmC- or 5fC-containing DNA fragment was prepared respectively by PCR amplification using dCTP, 5hmdCTP or 5fdCTP as substrate. The PCR products were digested with nuclease P1 to produce nucleotide mixtures containing either dCMP, 5hmdCMP or 5fdCMP, which further underwent deamination and dephosphorylation reactions. The relative abundance of the deamination products was determined in the forms of dU, 5hmdU or 5fdU (normalized to dC) from the incubations with or without DCTD or CDA. Three technical replicates were used to calculate the mean value. Data are mean ± standard error (mean).

***
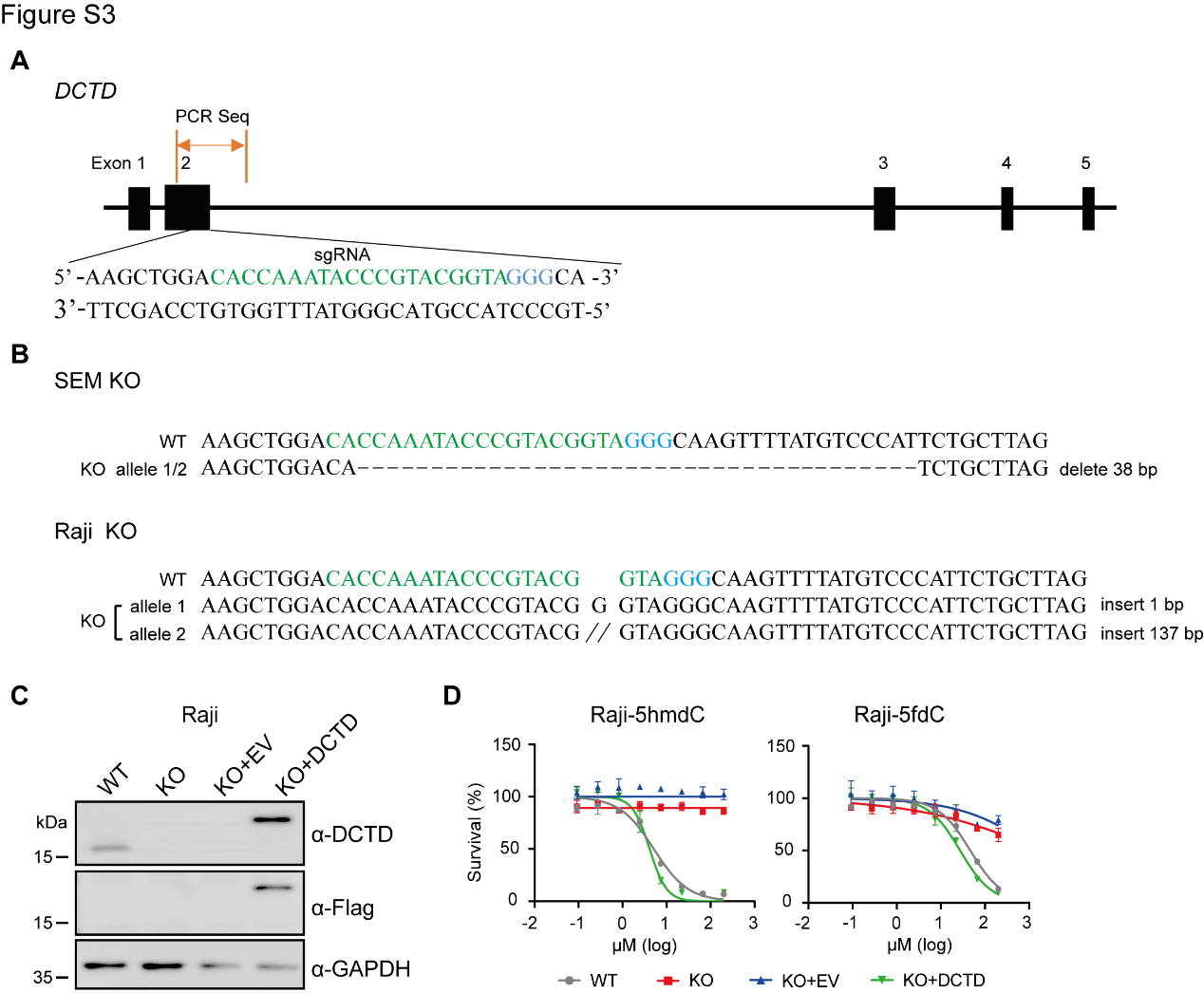
***

**Fig. S3. *DCTD* gene disruption mediated by CRISPR-Cas9 abolishes cytotoxicity of 5hmdC and 5fdC in Raji cells.**

(A) Sequence of sgRNA targeting exon 2 of *DCTD* gene is underlined in green, and the corresponding protospacer adjacent motif (PAM) sequence marked in blue. The region for PCR genotyping is indicated as horizontal arrows. (B) Indel (insertion/deletion) mutations detected in *DCTD* endogenous locus from the genome of SEM and Raji cells. sgRNA targeting sequences are labelled in green. PAM sequences are marked in blue. (C) Western blot showing DCTD protein level in Raji cell line of wild type (WT), *DCTD* knockout (KO), KO infected with empty lentiviral vector (KO+EV) or with vector containing DCTD (KO+DCTD). GAPDH was used as loading control. (D) IC50 curves of 5hmdC (**left**) and 5fdC (**right**) in wild type (grey), *DCTD* KO (red), KO+EV (blue) and KO+DCTD (green) Raji cells. X axis is the logarithmic transformed concentration of nucleosides. Y axis denotes the proportion of live cells on day 4 at each nucleoside concentration. Each dot represents the average value of 3 technical replicates. Data are mean ± standard deviation.


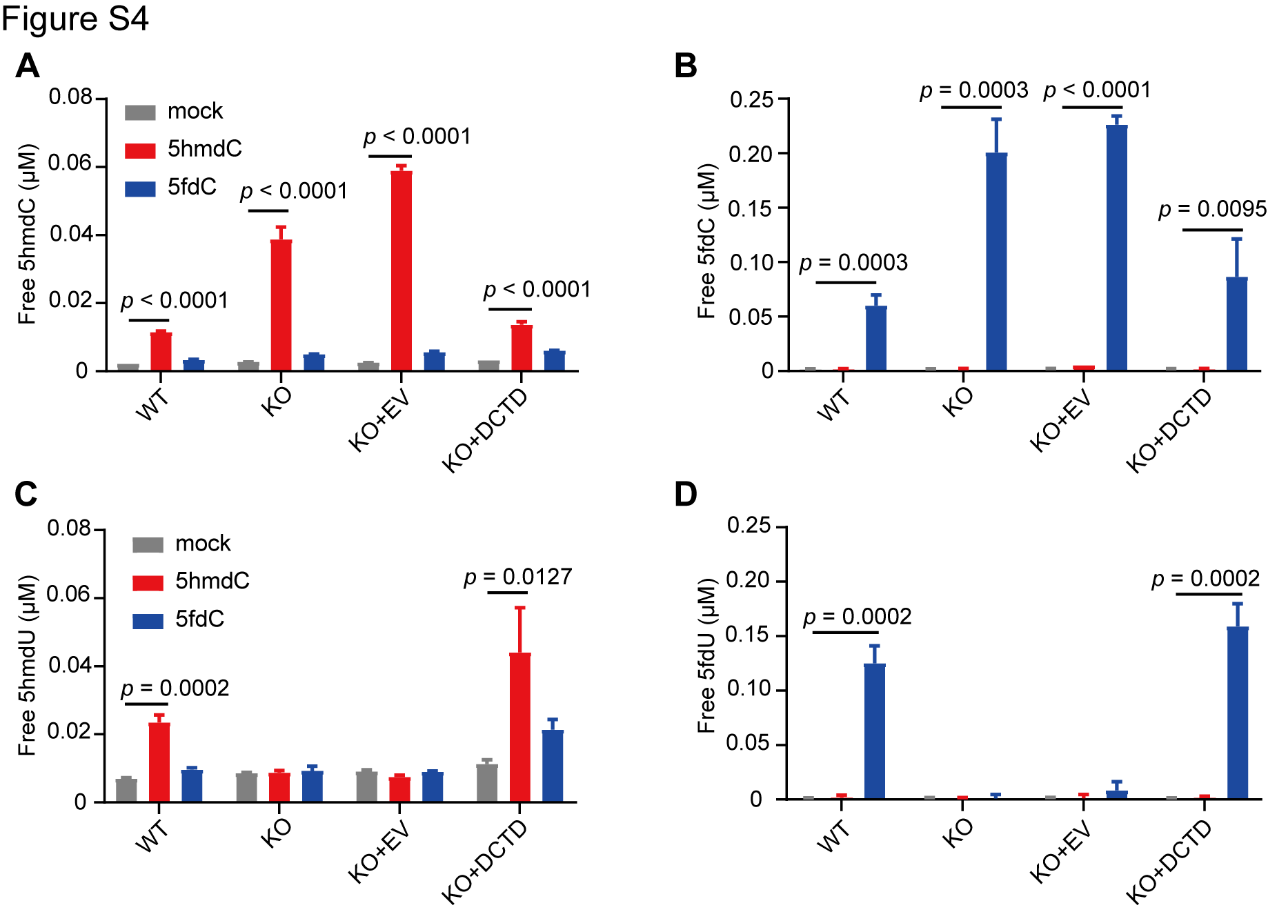


**Fig. S4. Detection of 5hmdC, 5fdC, 5hmdU and 5fdU nucleosides in cell metabolites of SEM cells by LC-MS/MS.**

(A-B) LC-MS/MS analysis of 5hmdC (A) and 5fdC (B) in the cellular metabolites of cell lines of wild type (WT), *DCTD* knockout (KO), KO infected with empty lentiviral vector (KO+EV) or with vector containing DCTD (KO+DCTD). Each cell line was treated in triplicate with PBS (mock), 10 μM 5hmdC or 5fdC. Data are mean ± standard error (mean). Significance is defined as *p* < 0.05. (C-D) LC-MS/MS analysis of 5hmdU (C) and 5fdU (D) in the cellular metabolites of cell lines of wild type (WT), *DCTD* knockout (KO), KO infected with empty lentiviral vector (KO+EV) or with vector containing DCTD (KO+DCTD). Each cell line was treated in triplicate with PBS (mock), 10 μM 5hmdC or 5fdC. Data are mean ± standard error (mean). Significance is defined as *p* < 0.05.

***
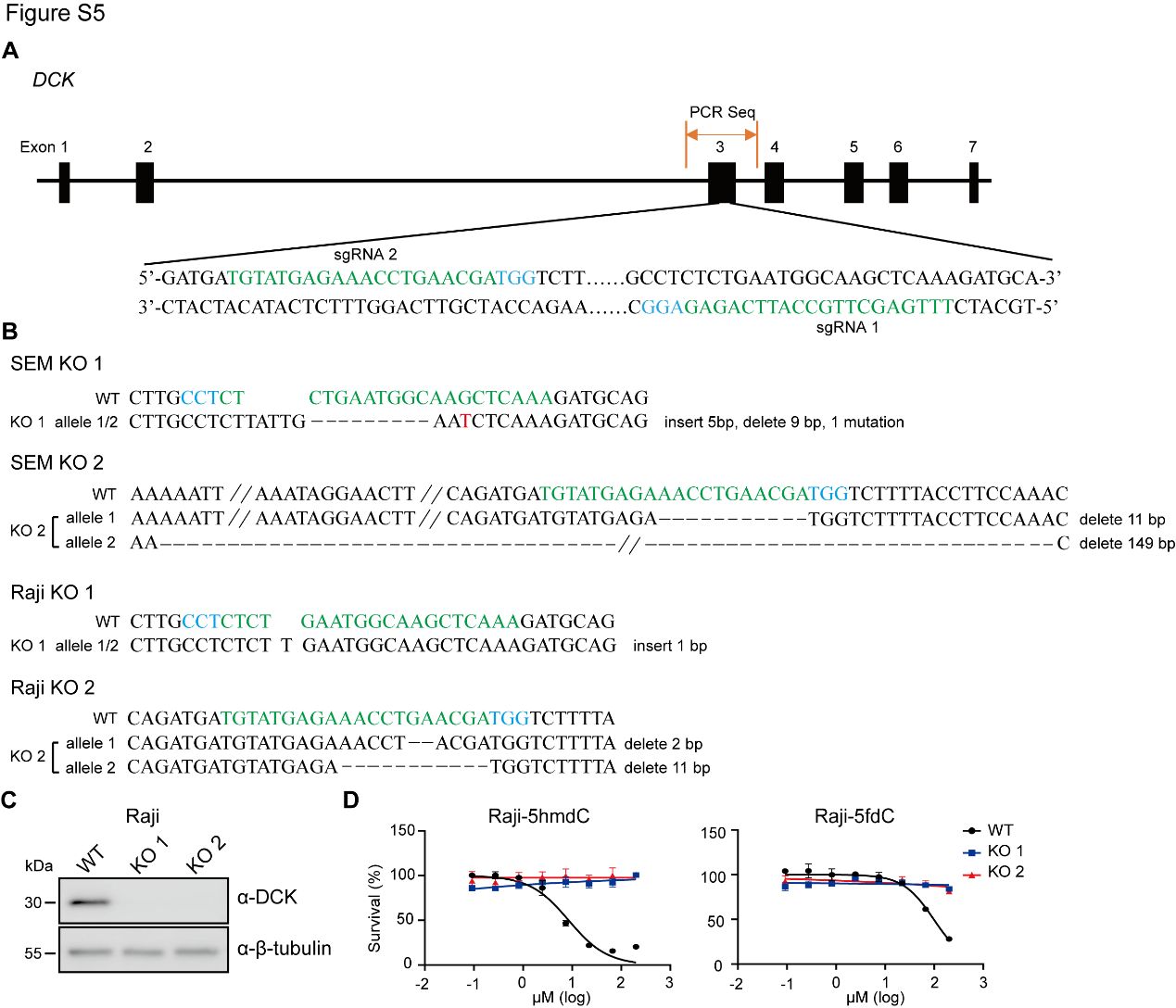
* Fig. S5. *DCK* gene disruption mediated by CRISPR-Cas9 abolishes cytotoxicity of 5hmdC and 5fdC in Raji cells.**

(A) Sequence of sgRNAs targeting exon 3 of *DCK* gene are in green, and the corresponding protospacer adjacent motif (PAM) sequence is marked in blue. The region for PCR genotyping is indicated above. (B) Indel (insertion/deletion) mutations detected at the *DCK* locus in the genomic DNA of SEM and Raji cells. sgRNA targeting sequences are labelled in green. PAM sequences are marked in blue. (C) Western analysis of DCK in wild type (WT) and two *DCK* knockout (KO 1 and KO 2) cell lines of Raji. Beta-tubulin was used as loading control. (D) IC50 curves of 5hmdC (**left**) and 5fdC (**right**) in WT and *DCK* KO Raji cell lines. X axis is the logarithmic transformed concentration of nucleosides. Y axis denotes the proportion of live cells on day 4 at each nucleoside concentration. Each dot represents the average value of 3 technical replicates. Data are mean ± standard error (mean).


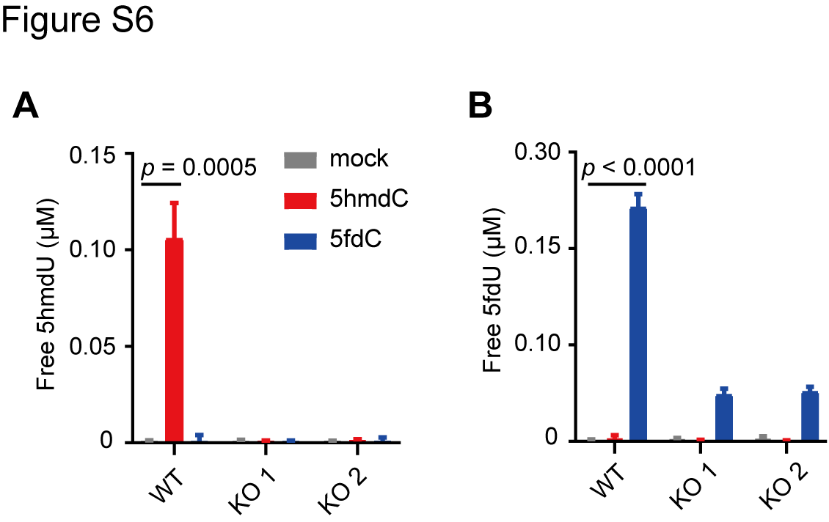


**Fig. S6. Detection of 5hmdU and 5fdU nucleosides in cell metabolites of SEM cells by LC-MS/MS.**

(A-B) Abundance of 5hmdU (A) and 5fdU (B) measured by LC-MS/MS in the cellular metabolites of WT and two independent *DCK* KO SEM cell lines treated in triplicate with PBS (mock), 10 μM 5hmdC or 5fdC. Total amount of genomic dC was used for normalization. Data are mean ± standard error (mean). Significance is defined as *p* < 0.05.

***
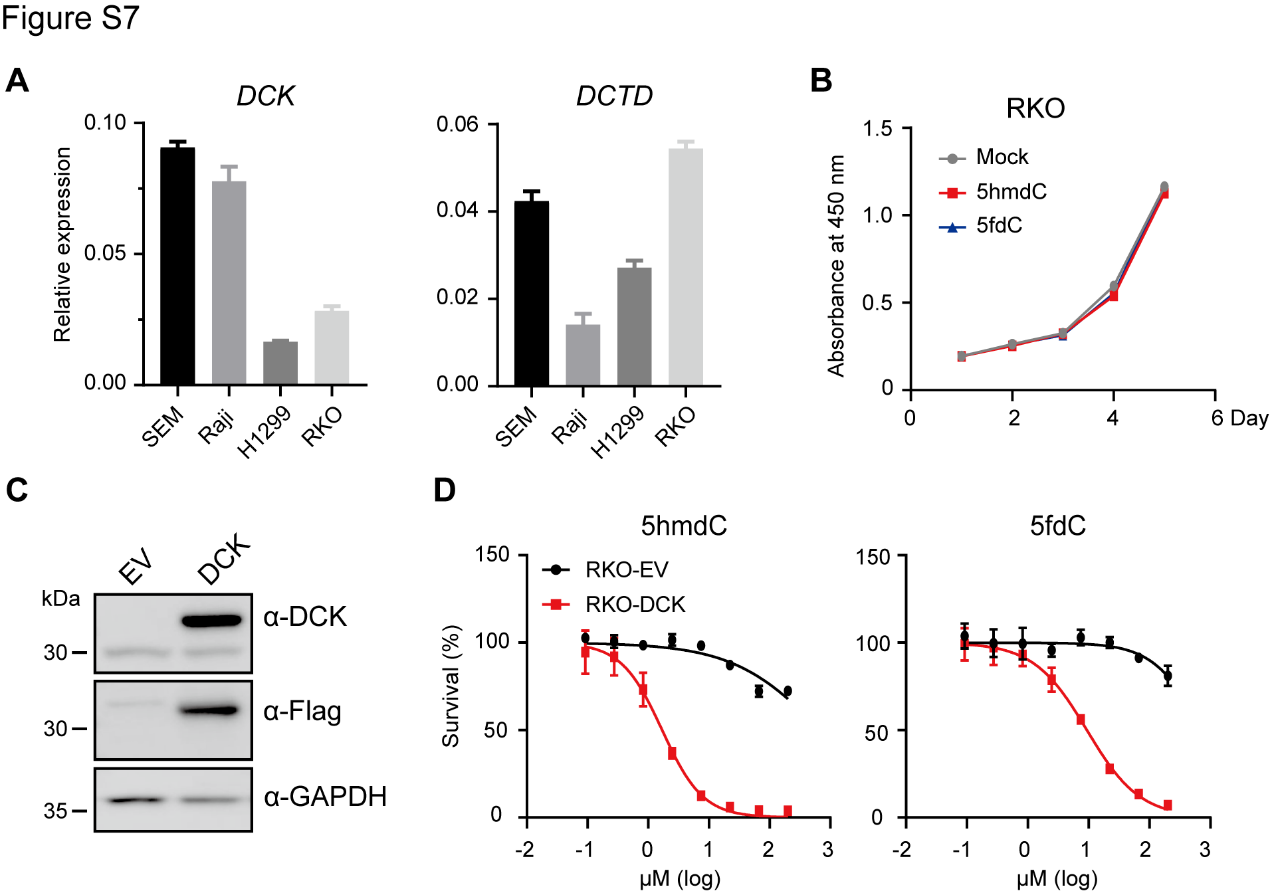
* Fig. S7. Ectopic expression DCK increases the sensitivity of RKO cells to 5hmdC and 5fdC.**

(A) mRNA level of *DCK* and *DCTD* in SEM, Raji, H1299 and RKO cell lines measured by RT-qPCR. *GAPDH* was used as the internal control. (B) Growth curves of RKO cell lines treated with PBS (mock, grey), 10 μM 5hmdC (red) or 5fdC (blue) over a period of 5 days. Each dot represents the mean value of three replicates. Y axis denotes the absorbance at 450 nm. Data are mean ± standard error (mean). (C) Western blot confirming DCK overexpression in RKO cell line. EV, RKO infected with empty lentiviral vector. DCK, RKO infected with Flag-DCK. GAPDH was used as loading control. (D) IC50 curves of 5hmdC (**left**) and 5fdC (**right**) in EV and DCK-overexpressing RKO cell lines. X axis is the logarithmic transformed concentration of nucleosides. Y axis denotes the proportion of live cells on day 4 at each nucleoside concentration. Each dot represents the mean value of three replicates. Data are mean ± standard error (mean).

***
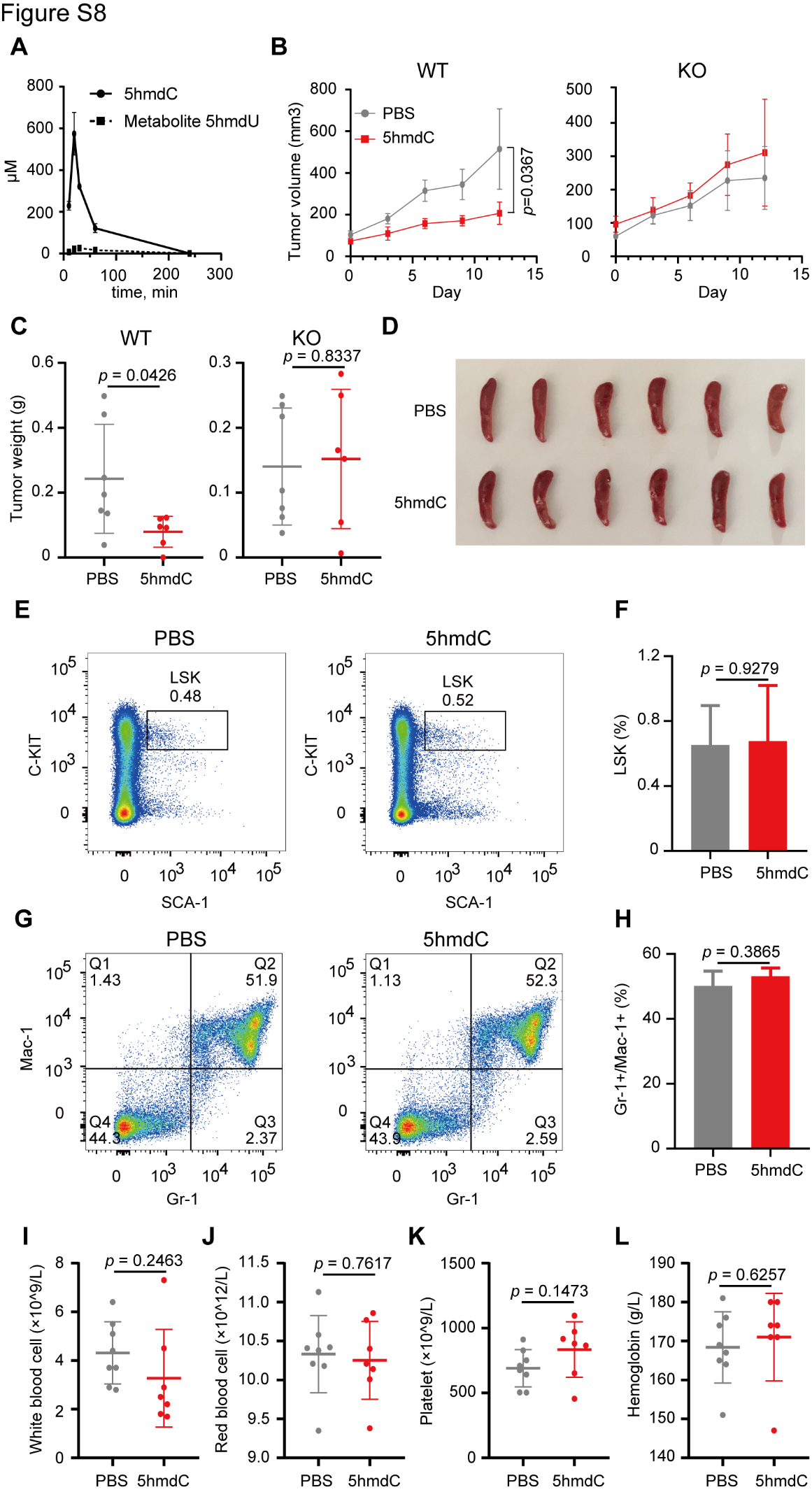
***

**Fig. S8. Evaluation of the physiological effects of 5hmdC in nude mice.**

(A) Pharmacokinetic profile of 5hmdC (50 mg/kg, intraperitoneal injection). Vein blood of the orbit was collected 10 min, 20 min, 30 min, 1 hour and 4 hours after 5hmdC administration. Y axis denotes the plasma concentration of 5hmdC or 5hmdU at each time point. X axis denotes the time of sample collection. (B) Tumor volumes of wild type (WT) and *DCTD* knockout (KO) Raji cells in BALB/c nude mice treated with PBS (n = 7) or 5hmdC (n = 6) were recorded every three days. Two-way repeated ANOVA analysis was used to compare the growth curve between PBS and 5hmdC groups. Significance is defined as *p* < 0.05. (C) Tumor weights of WT and *DCTD* KO Raji cells in BALB/c nude mice treated with PBS (n = 7) or 5hmdC (n = 6) at 50 mg/kg per day for eleven days. Each dot represents a single tumor. (D) Comparison of spleen size and morphology between PBS- and 5hmdC-treated group. Shown are spleens isolated from each group. (E-F) Percentage of Lin^−^Sca-1^+^c-Kit^+^ (LSK) hematopoietic stem cell population in PBS- and 5hmdC-treated mice analyzed by FACS (n = 3). Shown are representative plots of each group after eleven days of administration (E) and quantification of the LSK population in quadrant 2 (F). (G-H) Percentage of myeloid cell population (Gr-1^+^Mac-1^+^) in PBS- and 5hmdC-treated mice analyzed by FACS (n = 3). Shown are representative plots of each group after eleven days of administration (G) and quantification of the myeloid cell population in quadrant 2 (H). Data are mean ± standard error (mean). (I-L) Plasma concentrations of white blood cells (I), red blood cells (J), platelets (K) and hemoglobin (L) in peripheral blood of nude mice measured by a Mindray BC-2800Vet device after treatment with PBS or 5hmdC for eleven days.

***
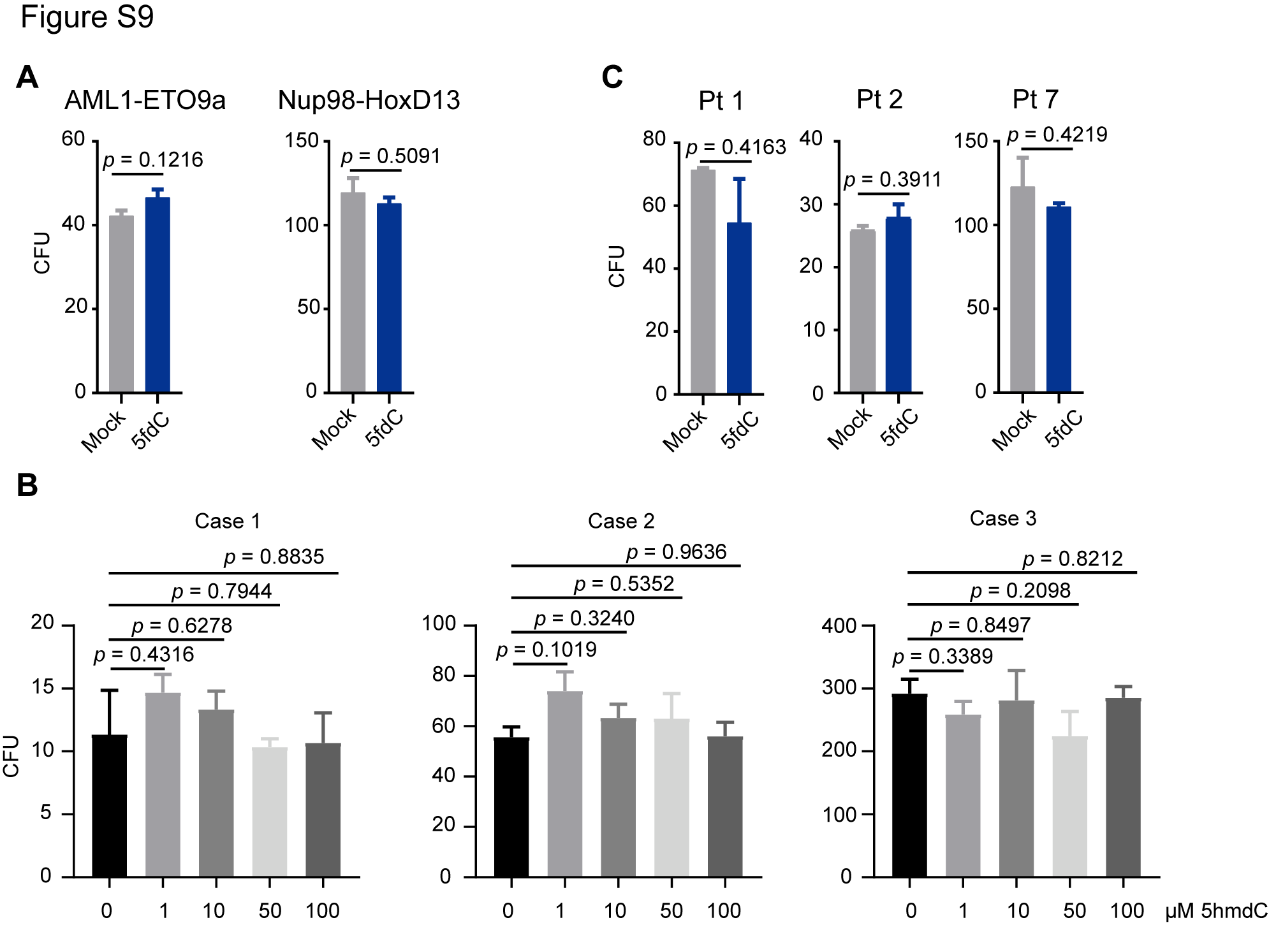
*Fig. S9.** **Colony formation assay.**

(A) Colony formation assay of bone marrow cells derived from mouse leukemia models harboring the fusion oncoproteins AML1-ETO9a or Nup98-HoxD13. Cells were cultured in triplicate in the presence of 10 μM 5fdC over a period of one week. Y axis denotes the counts of colony in each treatment group. Mock, cells treated with PBS. Data are mean colony number ± standard error (mean). Significance is defined as *p* < 0.05. (B) Colony formation assay of normal cord blood hematopoietic stem cells (CD34^+^). Cells were cultured with 0, 1, 10, 50, 100 μM 5hmdC in triplicate over a period of two weeks and numbers of colonies were scored. Y axis denotes the counts of colony in each treatment group. (C) Colony formation assay of bone marrow cells derived from leukemia patients. Cells were cultured with 20 μM 5fdC in triplicate over a period of two weeks and the numbers of colonies were scored. Y axis denotes the counts of colony in each treatment group. Pt, patient. For all panels: Mock, cells treated with PBS. Data are mean colony number ± standard error (mean). Significance is defined as *p* < 0.05.

***
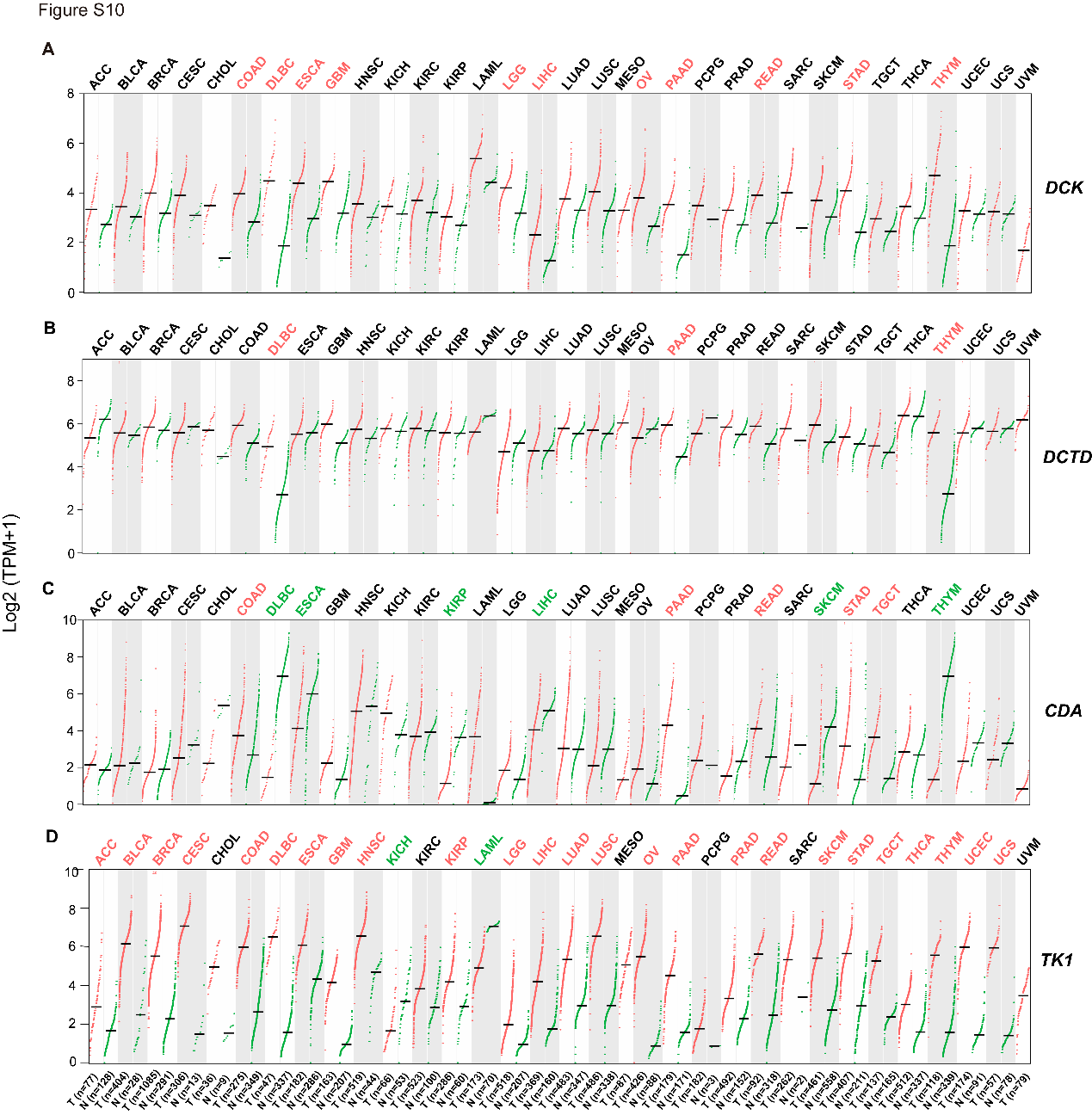
* Fig. S10. Expression of *DCK*, *DCTD*, *CDA* and *TK1* across multiple cancer types.**

RNA-seq data of *DCK* (A), *DCTD* (B), *CDA* (C) and *TK1* (D) was acquired from GEPIA interactive web server containing tumor and normal samples from the TCGA and the GTEx project. Each dot represents a distinct tumor or normal sample. Red font of tumor symbols on top of each panel represents significant upregulation in tumor tissues while green font represents downregulation. X axis denotes the sample numbers of tumor (T) and paired normal (N) tissues. Y axis is the logarithmic transformed expression data. Statistical difference was determined by ANOVA. TPM, transcripts per million. ACC, Adrenocortical carcinoma. BLCA, Bladder Urothelial Carcinoma. BRCA, Breast invasive carcinoma. CESC, Cervical squamous cell carcinoma and endocervical adenocarcinoma. CHOL, Cholangio carcinoma. COAD, Colon adenocarcinoma. DLBC, Lymphoid Neoplasm Diffuse Large B-cell Lymphoma. ESCA, Esophageal carcinoma. GBM, Glioblastoma multiforme. HNSC, Head and Neck squamous cell carcinoma. KICH, Kidney Chromophobe. KIRC, Kidney renal clear cell carcinoma. KIRP, Kidney renal papillary cell carcinoma. LAML, Acute Myeloid Leukemia. LGG, Brain Lower Grade Glioma. LIHC, Liver hepatocellular carcinoma. LUAD, Lung adenocarcinoma. LUSC, Lung squamous cell carcinoma. MESO, Mesothelioma. OV, Ovarian serous cystadenocarcinoma. PAAD, Pancreatic adenocarcinoma. PCPG, Pheochromocytoma and Paraganglioma. PRAD, Prostate adenocarcinoma. READ, Rectum adenocarcinoma. SARC, Sarcoma. SKCM, Skin Cutaneous Melanoma. STAD, Stomach adenocarcinoma. TGCT, Testicular Germ Cell Tumors. THCA, Thyroid carcinoma. THYM, Thymoma. UCEC, Uterine Corpus Endometrial Carcinoma. UCS, Uterine Carcinosarcoma. UVM, Uveal Melanoma.

**Supplementary Tables**

**Supplementary Table 1. IC50 of 5hmdC and 5fdC and mRNA levels of *DCTD*, *DCK* and *CDA* for 45 different cancer cell lines.**

| **Cell line** | **IC50 (μM)** | |  | **mRNA level (*10^-3^) (Relative to GAPDH)** | | |
| --- | --- | --- | --- | --- | --- | --- |
|  | **5hmdC** | **5fdC** |  | ***DCK*** | ***DCTD*** | ***CDA*** |
| SEM | 1.9780 | 5.9880 |  | 51.4 (2.66) ^b^ | 24.87 (0.92) | 0 (0) ^c^ |
| CCRF-CEM | 4.9770 | 1.9830 |  | 35.67 (1.75) | 9.19 (0.05) | 0 (0) |
| Raji | 16.6600 | 49.1000 |  | 40.99 (3.99) | 9.24 (0.9) | 0 (0) |
| TMD8 | 28.4300 | 405.6000 |  | 40.06 (2.29) | 28.49 (2.22) | 0 (0) |
| KG-1a | 31.5600 | 49.8100 |  | 50.93 (2.77) | 28.14 (1.87) | 0 (0) |
| 786-O | 34.2500 | 3915.0000 |  | 11.93 (0.5) | 7.44 (0.05) | 0 (0) |
| SK-N-SH | 208.3000 | 39408.0000 |  | 12.32 (1.11) | 14.69 (0.67) | 0 (0) |
| IMR-32 | 506.8000 | 218.7000 |  | 17.22 (2.09) | 20.55 (2.13) | 0 (0) |
| NCI-H1299 | 1198.0000 | 467.9000 |  | 7.81 (0.54) | 14.3 (1.52) | 0 (0) |
| Jurkat | 1382.0000 | 267.6000 |  | 36.64 (2.78) | 30.32 (2.41) | 0 (0) |
| LNCaP | ∞^a^ | 478.2000 |  | 26.49 (1.61) | 26.39 (1.93) | 0 (0) |
| SK-MEL-28 | ∞ | 237.5000 |  | 14.53 (0.97) | 14.34 (1.03) | 0 (0) |
| RKO | 3114.0000 | 1737.0000 |  | 15.57 (0.79) | 32.13 (0.78) | 0 (0) |
| HEL | 28309.0000 | 176.9000 |  | 28.45 (1.58) | 51.87 (2.52) | 0 (0) |
| K-562 | ∞ | 3976.0000 |  | 16.04 (0.62) | 27.89 (1.96) | 0 (0) |
| CEM/C1 | 11.2800 | 0.8738 |  | 36.91 (0.26) | 15.59 (0.06) | 0 (0) |
| NCI-H716 | 2490.0000 | 255.3000 |  | 16.8 (0.76) | 25.01 (1.58) | 0.01 (0) |
| U-87MG | ∞ | ∞ |  | 12.56 (1.94) | 18.91 (1.9) | 0.02 (0) |
| NCIH460 | ∞ | ∞ |  | 4.71 (0.2) | 12.26 (0.37) | 0.02 (0.01) |
| H9 | ∞ | 1526.0000 |  | 26.64 (3.13) | 45.82 (4.7) | 0.02 (0.02) |
| Hep 3B2.1-7 | 342.4000 | 203.1000 |  | 37.09 (1.26) | 25.43 (1.93) | 0.04 (0.01) |
| HS294T | 25.6100 | 325.4000 |  | 8.04 (0.45) | 16.11 (0.42) | 0.04 (0.01) |
| THP1 | 76.1000 | 2.9640 |  | 79.85 (9.61) | 46.61 (4.05) | 0.06 (0.01) |
| Calu6 | 77.0700 | 13.0100 |  | 10.12 (0.39) | 19.71 (2.61) | 0.06 (0) |
| NCI-H2170 | 1893.0000 | 32.8000 |  | 8.96 (0.47) | 12.37 (0.96) | 0.06 (0) |
| HCT-15 | ∞ | 1158.0000 |  | 18.45 (1.55) | 20.96 (1.81) | 0.1 (0) |
| ishikawa | 61.3800 | 98.0800 |  | 14.96 (1.31) | 40.67 (3.34) | 0.15 (0.01) |
| Kasumi-1 | 16236.0000 | 6122.0000 |  | 49.38 (4.15) | 58.81 (7.89) | 0.18 (0.01) |
| NCI-H661 | ∞ | 1280.0000 |  | 48.19 (2.75) | 54.69 (5.04) | 0.22 (0.02) |
| HT-144 | 773.6000 | 9170.0000 |  | 10.36 (1.22) | 19.21 (2.48) | 0.23 (0.03) |
| G-401 | 2384.0000 | 991.9000 |  | 8.99 (0.56) | 24.85 (1.88) | 0.25 (0.02) |
| U-2 OS | 188.8000 | 59.8000 |  | 17.23 (0.9) | 15.72 (1.05) | 0.28 (0.02) |
| OCI-AML3 | 85.8500 | 11.8200 |  | 14.59 (0.79) | 21.36 (0.93) | 0.34 (0.06) |
| RL95-2 | 7027.0000 | 6.1230 |  | 30.13 (1.26) | 67.83 (3.77) | 0.49 (0.03) |
| SKM1 | 2253.0000 | 4.8740 |  | 106.83 (1.13) | 64.59 (2.46) | 0.54 (0.09) |
| HL-60 | ∞ | ∞ |  | 22.89 (0.95) | 27.52 (0.58) | 0.54 (0.04) |
| HEC-1-A | 60364.0000 | 28.9800 |  | 31.7 (1.01) | 47.75 (7.55) | 0.61 (0.05) |
| HT-29 | 31500.0000 | 45.5800 |  | 13.7 (0.44) | 57.28 (2.44) | 0.8 (0.04) |
| calu-3 | ∞ | 41.9000 |  | 7 (0.37) | 18.39 (2.76) | 1.37 (0.38) |
| Caki-1 | 423.7000 | 1807.0000 |  | 7.29 (2.64) | 28.46 (4.31) | 1.93 (0.37) |
| Du 145 | ∞ | 6.1370 |  | 10.53 (0) | 13.28 (0.81) | 5.16 (0.13) |
| PC3 | 117.1000 | 2.2940 |  | 10.49 (1.65) | 23.55 (1.53) | 5.78 (0.6) |
| Sum149 | 2.8000 | 2.8830 |  | 10.45 (0.1) | 27.49 (0.4) | 7.47 (0.11) |
| MDA-MB-231 | 24.7100 | 30.5200 |  | 22.46 (0.09) | 32.63 (2.41) | 8.73 (0.76) |
| HepG2 | ∞ | 34.4100 |  | 5.82 (1.03) | 5.63 (0.22) | 20.63 (0.86) |

^a^ ∞ indicates the IC50 is too high to be estimated. ^b^ Expression is given as mean (S.D.). ^c^ 0 indicates Ct > 35.

**Supplementary Table 3. Patient information and mutation profiles.**

| **Sample** | **Gender** | **Age** | **Diagnosis** | **Gene mutations** |  |
| --- | --- | --- | --- | --- | --- |
| Patient 1 | Male | 38 | Acute myeloid leukemia | *DNMT3A*: c.2644C>A (p.R882S), heterozygous |  |
|  |  |  |  | *TET2*: c.86C>G(p.P29R), heterozygous |  |
|  |  |  |  | *TET2*: c.5284A>G(p.I1762R), heterozygous |  |
|  |  |  |  | *TP53*: c.215C>G(p.P72R), heterozygous |  |
| Patient 2 | Female | 72 | Acute myeloid leukemia | *IDH2*: c.515G>A (p.R172K), heterozygous |  |
|  |  |  |  | *ASXL1*: c.1954G>A (p.G652S), heterozygous |  |
|  |  |  |  | *ASXL1*: c.3759T>C (p.S1253S), heterozygous |  |
|  |  |  |  | *DNMT3A*: c.2645G>C (p.R882P), heterozygous |  |
|  |  |  |  | *WT1*: c.1107A>G (p.R369R), heterozygous |  |
| Patient 3 | Male | 74 | Acute myeloid leukemia | *TET2*: c.86C>G(p.P29R), heterozygous |  |
|  |  |  |  | *TET2*: c.5284A>G(p.I1762V), heterozygous |  |
|  |  |  |  | *DNMT3A*: c.2256C>A(p.F752L), heterozygous |  |
|  |  |  |  | *TP53*: c.215C>G(p.P72R), homozygous |  |
|  |  |  |  | *WT1*: c.1107A>G(p.R369R), homozygous |  |
| Patient 4 | Female | 59 | Acute myeloid leukemia | Not available |  |
| Patient 5 | Female | 56 | Myelodysplastic syndrome | Not available |  |
| Patient 6 | Male | 54 | Acute myeloid leukemia | Not available |  |
| Patient 7 | Female | 80 | Acute myeloid leukemia | *TET2*: c.2585del(p.Leu862CysfsX11), heterozygous |  |
|  |  |  |  | *TET2*: c.5284A>G(p.I1762V), heterozygous |  |
|  |  |  |  | *ASXL1*: c.1934_1935insG(p.G645fsX13), heterozygous |  |
|  |  |  |  | *ASXL1*: c.3759T>C(p.S1253S), heterozygous |  |
|  |  |  |  | *TP53*: c.215C>G(p.P72R), heterozygous |  |
|  |  |  |  | *WT1*: c.1107A>G(p.R369R), heterozygous |  |
|  |  |  |  | *RUNX1*: c.777dupT(p.N260*), heterozygous |  |
|  |  |  |  | *RUNX1*: c.329A>G(p.K110R), heterozygous |  |
| Patient 8 | Male | 17 | Acute myeloid leukemia | *C-KIT*: c.1261_1262ins (p.L421Lfs*560), heterozygous | |
|  |  |  |  | *ASXL1*: c.1954G>A (p.G652S), heterozygous | |
|  |  |  |  | *TET2*: c.86C>G(p.P29R) , heterozygous |  |
| Patient 9 | Male | 71 | Acute myeloid leukemia | Not available |  |
| Patient 10 | Male | 15 | Acute myeloid leukemia | *TET2*: c.86C>G(p.P29R), heterozygous |  |
|  |  |  |  | *WT1*: c.1107A>G(p.R369R), heterozygous |  |
|  |  |  |  | *ASXL1*: c.3759T>C(p.S1253S), heterozygous |  |

**Methods and materials**

**Cell culture**

Cells were cultivated in suitable medium supplemented with 10% FBS and 1% penicillin/streptomycin (Invitrogen) at 37 °C at 5% CO_2_. HEK-293T cells were cultured in DMEM (11965092, Thermo Fisher Scientific). Raji, H1299 and RKO were grown in RPMI-1640 (22400071, Thermo Fisher Scientific); SEM was cultivated in IMDM (12440053, Thermo Fisher Scientific). *Eµ-Myc p19^Arf-/-^* mouse B cell lymphoma cells were cultured in B-cell medium as previously described (Jiang et al., 2020). Raji and SEM cell lines as well as bone marrow cells derived from mouse leukemia models harboring fusion oncoproteins AML1-ETO9a or Nup98-HoxD13 were provided by Lan Wang (Shanghai Institute of Nutrition and Health, CAS). HEK-293T, RKO and H1299 were obtained from the Shanghai Cell Bank of the Chinese Academy of Sciences (Shanghai, China). Cell proliferation assays were performed by seeding cells in 96-well plates with indicated concentrations of PBS, 5hmdC or 5fdC. Cell proliferation was measured by Cell Counting Kit-8 (CCK8) (Yeasen, 40203ES60) according to manufacturer’s instructions.

[**Half maximal inhibitory concentration (IC50)**](https://pubmed.ncbi.nlm.nih.gov/27492086/) **assay**

Cells were seeded at a density of 6000 cells (SEM, Raji) or 1000 cells (H1299, RKO) per well in triplicate in 96-well plates. Oxidized methylcytidines were serially diluted in PBS in a ratio of 1:3, generating 8 different concentrations ranging from 0 to 200 μM. The dilution series were added into 96-well plates. Untreated cells were used as positive controls, and media as a negative control. After a 4-day treatment, a CCK8 assay was performed according to the manufacturer’s protocol. IC50 was calculated using GraphPad Prism 8 software.

**CRISPR-Cas9 knockout screen**

Construction of the Cas9-expressing *Eµ-Myc p19^Arf-/-^* stable cell line and CRISPR dropout/enrichment screens were performed as previously described (Jiang et al., 2020). Cells were treated with 200 µM 5hmdC for two rounds, which kills 95% of uninfected cells. Bioinformatic analysis was conducted using the MAGeCK (Model-based Analysis of Genome-wide CRISPR-Cas9 Knockout) algorithm (Li et al., 2014).

**Antibodies**

Antibodies used were as follows. Rabbit anti-CDA (ab82346, Abcam), rabbit anti-DCK (ab96599, Abcam), mouse anti-DCTD (sc-376659, Santa Cruz Biotech), mouse anti-β-tubulin (86298S, Cell Signaling Technologies), rabbit anti-H2AX (7631S, Cell Signaling Technologies), rabbit anti-γH2AX (9718S, Cell Signaling Technologies), rabbit anti-Flag (F7425, Sigma Aldrich) and mouse anti-GAPDH (ab0037, Abways). The following antibodies were used for flow cytometry analysis: Ly-6A/E (Sca-1) (45-5981-82, Invitrogen), CD117 (c-Kit) (17-1172-82, ebioscience), TER-119 (11-5921-85, ebioscience), CD2 (11-0021-82, ebioscience), CD3 (11-0032-82, ebioscience), CD5 (14-0051-85, ebioscience), CD8a (11-0081-85, ebioscience), CD45R (B220) (11-0452-82, ebioscience), Ly-6G/Ly-6C (Gr-1) (11-5931-85, ebioscience), CD11b (Mac-1) (25-0112-81, ebioscience).

**Lentiviral packaging and CRISPR-Cas9-mediated gene disruption**

Single-guide RNA (sgRNA) oligonucleotides targeting *DCK* or *DCTD* were designed using GPP Web Portal (powered by Broad Institute) and were annealed and cloned into a LentiCRISPR v2 plasmid. Lentiviruses were produced by co-transfection of 293T cells with packaging plasmids psPAX2 and pMD2.G, plus either LentiCRISPR v2-sgRNA construct or pCDH-CMV-3 × Flag -EF1-copGFP. Cells were seeded together with viruses into a 12-well plate precoated with RetroNectin (T100A, TAKARA). The plate was centrifuged at 1000 × *g* for 90 min. GFP-positive cells were sorted and seeded into 96-well plates at a density of single cell per well. The knockout clones were confirmed by genotyping and western blot. The sgRNA sequences are as follows. *DCTD*-sgRNA-F, CACCGCACCAAATACCCGTACGGTA. *DCTD*-sgRNA-R, AAACTACCGTACGGGTATTTGGTGC. *DCK*-sgRNA-F1, CACCGTTTGAGCTTGCCATTCAGAG. *DCK*-sgRNA-R1, AAACCTCTGAATGGCAAGCTCAAAC. *DCK*-sgRNA-F2, CACCGTGTATGAGAAACCTGAACGA. *DCK*-sgRNA-R2, AAACTCGTTCAGGTTTCTCATACAC. Genotyping primers are as follows. *DCTD*-primer-F, GTTTTATTCCCTGCTTCCACAGCG. *DCTD*-primer-R, CACCATGCACATCCCTCAGC. *DCK*-primer-F, GATCTAAGGATTTTCCAGACCTCAGAC. *DCK*-primer-R, GGAGGTCAAAAGAATCCTAGCATAAGT.

**Expression and purification of human DCTD and CDA recombinant protein**

The open reading frame (ORF) lacking the initiation codon of *DCTD* (Gene ID: 1635) was cloned into modified pET28a (pPEI-His-SUMO, supplied by Yanhui Xu, Shanghai Medical College of Fudan University). The open reading frame (ORF) of CDA (Gene ID: 978) was cloned into pET28a. The expression and purification of DCTD and CDA were carried out as previously described (Xue et al., 2019). His-Tagged CDA protein was bound to Ni-NTA beads (Qiagen), which were washed with washing buffer (50 mM Tris-HCl, pH 7.5, 25 mM imidazole, 1 mM DTT and 10% glycerol). Target protein was eluted from the Ni-NTA beads by elution buffer (50 mM Tris-HCl, pH 7.5, 250 mM imidazole, 1 mM DTT and 10% glycerol), and then desalted by ultrafiltration. Proteins were analyzed on a 12% SDS-PAGE gel.

**Preparation of substrates for *in vitro* deamination assay**

A 490-bp C-, 5hmC- or 5fC-containing DNA fragment (C-DNA, 5hmC-DNA and 5fc-DNA) was prepared respectively by PCR amplification from a template DNA containing 41% G/C using dCTP (BIO-39038, Bioline), 5-hydroxymethy-dCTP (5hmdCTP, N-2060-1, Trilink) or 5-formyl-dCTP (5fdCTP, N-2064-1, Trilink) as substrate. Upon deamination, the C-DNA, 5hmC-DNA and 5fC-DNA fragments were subjected to nuclease P1 (Sigma) digestion to produce nucleotide mixtures containing either dCMP, 5hmdCMP or 5fdCMP respectively.

***In vitro* DCTD and CDA deamination assay**

A total of 0.05 μg of DCTD or CDA protein was incubated with 100 μM substrate (dCMP-, 5hmdCMP- or 5fdCMP-containing mixture) in 100 mM Tris-HCl (pH 7.0) for 20 minutes at 37 °C. Enzymes were inactivated at 98 °C for 3 min. After treatment with calf intestinal alkaline phosphatase (CIAP, Takara) for 4 h at 37 °C, variants of deoxyuridine and deoxycytidine were analyzed by LC-MS/MS.

**Western blot assay**

Cell pellets were resuspended in 100 μl RIPA lysis buffer (KGP702-100, Jiangsu KeyGEN BioTECH). Samples were subjected to ultrasonication for 5 min to disrupt the genomic DNA and then mixed with SDS loading buffer, followed by denaturation at 98 °C for 8 min. Protein separation and blotting were performed as previously described (Kim et al., 2017). Blots were developed with ECL detection reagent (180-5001, Tanon) and imaged on a CCD imager (GE Healthcare).

**Quantitative real-time PCR (RT-qPCR)**

Total cellular RNA was extracted from cell lines or primary leukemia cells using the Qiagen RNAeasy kit (74104, Qiagen). cDNA synthesis was performed using a cDNA Synthesis Kit (K1671, Thermo Fisher SCIENTIFIC) according to the manufacturer’s instructions. Quantitative real-time PCR was performed on a LightCycler 480Ⅱ(Roche) using Luna Universal qPCR Master Mix (M3003s, NEB). Expression levels were determined using the ΔΔCt method and normalized to the level of *GAPDH* or *Actb*. Three technical replicates were used to calculate the mean value. The primers for RT-qPCR are as follows. *CDA*-F, AAGTCAGCCTACTGCCCCTAC. *CDA*-R, GATAGCGGTCCGTTCAGCAC. *DCTD*-F, TGCAAGAAACGGGACGACTAT. *DCTD*-R, ATCACTGCACCCATTTGGCAT. *DCK*-F, CCATCGAAGGGAACATCGCT. *DCK*-R, GGTAAAAGACCATCGTTCAGGT. *DNPH1*-F, GCCGTGGCCTTTAACAAGC. *DNPH1*-R, GGAGGATCAGCCTCGAAGTATC. *GAPDH*-F, GGAGCGAGATCCCTCCAAAAT. *GAPDH*-R, GGCTGTTGTCATACTTCTCATGG. *Cda*-F, GATCTTCTCTGGGTGCAACATAG. *Cda*-R, CCTGAAATCCTTGTACCCTTCG. *Dctd*-F, TGAACAAGAACTCGGCTGATG. *Dctd*-R, CGCTGTCGTGGTATTTATCCG. *Dck*-F, GAAGAGCGGTGGAAATGTTCT. *Dck*-R, GCATCTTTGAGCTTGCCATTG. *Dnph1*-F, AGTAGCTCTTGGTAAGCCGAT. *Dnph1*-R, TTCTTCCTCTGCGTAGTCCCA. *Actb*-F, GGCTGTATTCCCCTCCATCG. *Actb*-R, CCAGTTGGTAACAATGCCATGT.

**Colony-forming assay**

Human primary bone marrow cells derived from leukemia patients were obtained at Huashan Hospital of Fudan University. Mononuclear cells were collected by density gradient centrifugation with Ficoll (1077, Sigma). CD34^+^ cells were isolated from human cord blood as previously described (Guo et al., 2017). Human cells were seeded in triplicate using MethoCult Medium (H4434, STEMCELL Technologies) supplemented with 20 μM 5hmdC or 5fdC. Mouse bone marrow cells were seeded in triplicate in MethoCult Medium (M3434, STEMCELL Technologies) supplemented with 10 μM 5hmdC or 5fdC. Cells were cultivated at 37°C at 5% CO_2_ for one (mouse leukemia cells) or two weeks (patient samples), and numbers of colonies were scored.

**Fluorescence-activated cell sorting (FACS)**

Flow cytometry analysis was performed on a BD FACS Canto Ⅱ (BD Biosciences) and data were analyzed with FlowJo software. Cell sorting was performed on a FACS Aria Ⅲ (BD Biosciences).

**Extraction of genomic DNA and cell metabolites**

The genomic DNA was extracted with phenol-chloroform and digested into nucleosides as previously described (He et al., 2011). Proteins were removed by Nanosep Centrifugal Devices with Omega Membrane 3K (PALL). For cell metabolites, cells were collected and washed in ice-cold PBS. Cell pellets were suspended in hypotonic buffer (10 mM Tris-HCl, pH 8.0, 1mM KCl, 1.5 mM MgCl_2_, and 1 mM DTT) and passed through an insulin syringe ten times, followed by centrifugation at 14,000 rpm at 4 °C for 15min. The supernatant was mixed with methyl alcohol and then shaken at 2,000 rpm at 4 °C for 20min. Insoluble material was pelleted and the supernatants were used for composition analysis. The concentration of intracellular free nucleosides is calculated using the average cell volume value of 2.6 ml/10^9^ cells (Traut, 1994).

**Nucleoside analysis by LC-MS/MS**

To quantitatively determine the nucleoside contents in the genome and cell metabolites, a HPLC system (Ultimate 3000, Thermo Fisher Scientific) coupled with a triple quadrupole mass spectrometer (QTRAP 6500, AB SCIEX) was used for LC-MS/MS analysis. Chromatography was performed by an ACQUITY UPLC HSS T3 column (1.8 μm; 2.1 mm × 150 mm, Waters) at 45 °C. The mobile phases (A, 0.075% formic acid in 100% water; B, 0.075% formic acid in 100% acetonitrile) were used for compound separation at a flow rate 0.2 ml/min: 100 % A (0–3 min), 100-85% A (3–5 min), 85 to 60% A (5–8 min), 60 to 20% A (8–9 min), 20% A (9–14 min), 20-100% A (14–15 min), 100% A (15–25 min) for re-equilibrium. All compounds were measured on ESI-positive mode. The selective MRM transitions were monitored as follows: dC (228.0/112.0), 5hmdC (258.0/142.0), 5fdC (256.1/140.1), 5hmdU (257.0/124.1) and 5fdU (255.0/212.0). The raw mass spectrometry data were analyzed using the Skyline-daily software.

**Subcutaneous xenografts and blood tests**

Female BALB/c nude mice (5-week-old) were purchased from the Charles River (Beijing, China) and cared for in accordance with the National Institutes of Health Guide for the Care and Use of Laboratory Animals. All experimental work was approved in advance by the Ethics Review Committee for Animal Experimentation of Fudan University. Subcutaneous xenograft and administration of 5hmdC were performed as previously described (Zauri et al., 2015). Animals were intraperitoneally (i.p.) injected with PBS or 50 mg/kg 5hmdC daily. Tumor volume was calculated with the following formula: L*W*W/2, in which L and W represent the length and width of the tumor. Vein blood of orbit from three mice at each timepoint was collected for nucleoside concentration measurement. The complete blood count was analyzed on a Mindray BC-2800Vet device.

**Immunohistochemistry**

Xenograft tumors were dissected and fixed in 4% paraformaldehyde for 24 h before being sectioned for immunostaining. The TUNEL assay and γH2AX immunostaining were performed as previously described (Kim et al., 2017). Photomicrographs were captured on an Olympus DP74 microscope.

**Statistical analysis**

All values are expressed as mean ± standard error (mean). Statistical significance was evaluated by two-way repeated ANOVA analysis for cell and tumor growth curves or two-tailed Student’s *t*-tests for all others, using GraphPad Prism 8. The statistical significance levels were set at **p* < 0.05, ***p* < 0.01 and ****p* < 0.001. ANOVA was used to determine the statistical difference between tumor and paired normal samples acquired from GEPIA interactive web server. The cutoffs of Log2FC and q-value are set at 1 and 0.01, respectively.

**Reference**

Guo, B., Huang, X., Cooper, S., and Broxmeyer, H.E. (2017). Glucocorticoid hormone-induced chromatin remodeling enhances human hematopoietic stem cell homing and engraftment. Nat Med 23, 424-428.

He, Y.F., Li, B.Z., Li, Z., Liu, P., Wang, Y., Tang, Q.Y., Ding, J.P., Jia, Y.Y., and Chen, Z.C. (2011). Tet-Mediated Formation of 5-Carboxylcytosine and Its Excision by TDG in Mammalian DNA. Science 333, 1303-1307.

Jiang, W., Yang, A., Ma, J., Lv, D., Liu, M., Xu, L., Wang, C., He, Z., Chen, S., Zhao, J.*, et al.* (2020). Genetic determinants of COVID-19 drug efficacy revealed by genome-wide CRISPR screens. bioRxiv.

Kim, J., Hu, Z., Cai, L., Li, K., Choi, E., aubert, B.F., Bezwada, D., Rodriguez-Canales, J., Villalobos, P., and Lin, Y.F. (2017). CPS1 maintains pyrimidine pools and DNA synthesis in KRAS/LKB1-mutant lung cancer cells. Nature 546, 168-172.

Li, W., Xu, H., Xiao, T., Cong, L., Love, M.I., Zhang, F., Irizarry, R.A., Liu, J.S., Brown, M., and Liu, X. (2014). MAGeCK enables robust identification of essential genes from genome-scale CRISPR/Cas9 knockout screens. Genome Biology 15, 554-565.

Traut, T.W. (1994). Physiological concentrations of purines and pyrimidines. Molecular&Cellular Biochemistry 140, 1-22.

Xue, J.H., Chen, G.D., Hao, F., Chen, H., Fang, Z., Chen, F.F., Pang, B., Yang, Q.L., Wei, X., and Fan, Q.Q. (2019). A vitamin-C-derived DNA modification catalysed by an algal TET homologue. Nature 569, 581-585.

Zauri, M., Berridge, G., Thézénas, M.L., Pugh, K.M., Goldin, R., Kessler, B.M., and Kriaucionis, S. (2015). CDA directs metabolism of epigenetic nucleosides revealing a therapeutic window in cancer. Nature 524, 114-118.
